# Decarbonizing the emirates: A roadmap to net-zero emissions by 2050 in the UAE

**DOI:** 10.1016/j.isci.2025.114348

**Published:** 2025-12-08

**Authors:** Roghayeh Yousef, Niall Mac Dowell

**Affiliations:** 1 Centre for Environmental Policy, Imperial College London, London SW7 1NE, UK; 2 The Sargent Centre for Process Systems Engineering, Imperial College London, London SW7 2AZ, UK

**Keywords:** energy modeling, energy policy, energy resources, energy sustainability, energy systems

## Abstract

Recognized as a major global energy exporter, the United Arab Emirates (UAE) faces the unique challenge of transitioning to more environmentally sustainable practices while maintaining its pivotal role in global energy supply. Despite an abundance of literature focusing on the transition of net-importer countries toward carbon neutrality, limited attention has been given to the socially and economically viable pathways of energy net-exporter countries. This study examines the UAE’s transition toward carbon neutrality across the power, industrial, and transport sectors. It highlights variations in technology deployment and emphasizes the importance of prioritizing domestic biomass and investing in technologies like direct air capture (DAC). The findings highlight the broader significance of collaborative efforts to advance sustainable energy practices not only in the UAE but also globally.

## Highlights

• Imported biomass enables BECCS; domestic biomass requires DAC and +25 GW
• Electrified LNG and industrial CCS scale by 2035
• Full road EV adoption by 2050 avoids £25bn in additional costs
• Decarbonization costs +9.4% vs. BAU; ∼1.3% of UAE’s annual GVA

## Graphical abstract

**Figure:**
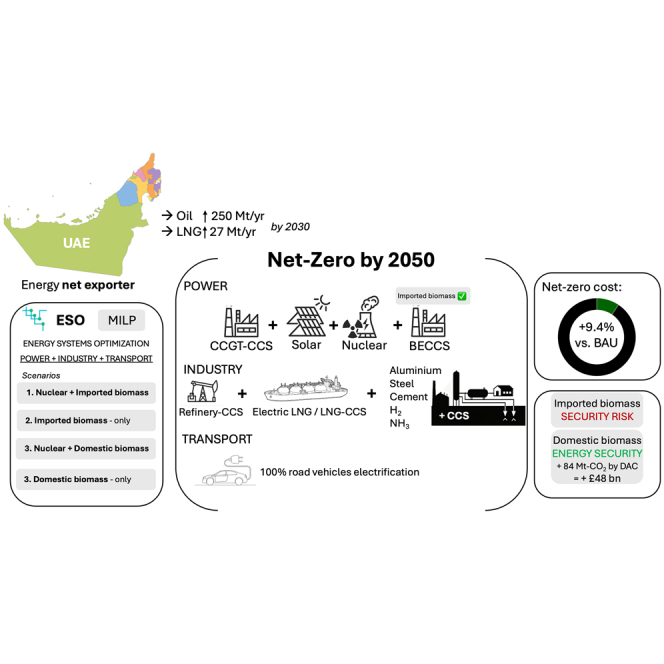

## Introduction

Recognized as a major global energy exporter, the United Arab Emirates (UAE) supplies 3.2% of the world’s oil demand.^1^ As the first country in the MENA (Middle East and North Africa) region to pledge achieving net-zero emissions by 2050,^2^ the UAE faces a unique challenge similar to other net-exporter countries worldwide.^3^^,^^4^ It must balance its status as a leading energy exporter^5^^,^^6^ with the imperative of transitioning to more environmentally sustainable practices. This balance is critical, as many countries rely on the UAE’s energy exports to sustain their economies. Notably, India imports 7% of its oil needs from the UAE,^7^ while Bangladesh imports 23% of its oil from both the UAE and Saudi Arabia.^8^ Both nations have their own net-zero targets, with India aiming for nationwide net-zero by 2070^9^ and Bangladesh targeting 2050 in Dhaka.^10^ Given the heavy reliance of many nations on the UAE’s energy exports,^11^^,^^12^^,^^13^ critical questions arise regarding how the UAE can achieve its environmental goals without compromising its energy supply to these net-importer countries and hindering their paths toward net-zero. Abrupt changes are not a realistic approach; instead, a gradual transition to lower carbon intensity is key to ensuring a smooth transition for both the UAE and the countries reliant on its energy exports.

Given the scale of its oil production and associated carbon emissions, the UAE’s transition to net-zero is not only essential for meeting its national commitments but also for demonstrating how major fossil fuel-exporting countries can achieve carbon neutrality while maintaining their economic roles. This makes the UAE an ideal test bed for exploring how net energy-exporting countries can formulate and implement a roadmap for decarbonization. Developing a comprehensive and technically feasible roadmap for the UAE is crucial to providing replicable insights that other fossil fuel exporters can leverage in their own transitions.

The global commitment toward carbon neutrality is multifaceted, with most initial pledges originating from net-importer countries, followed subsequently by net-exporters. This precedence has shaped recent literature, which has extensively focused on how net-importers can transition toward carbon neutrality, often overlooking the impact of net-exporter economies on this transition.^14^^,^^15^ One significant example preceding this phenomenon was the Russian-Ukrainian war, which compelled Germany to postpone its coal decommissioning plans.^16^ Subsequent studies have highlighted the imperative for carbon capture and storage (CCS) in Germany to facilitate medium-term blue hydrogen production and its integration into coal capacities.^17^^,^^18^ Before this shift, Germany’s transition strategy predominantly favored renewable and bio-energy sources^19^^,^^20^ in the literature. However, certain earlier projections still considered the continued use of fossil energy by 2050,^21^^,^^22^ which is no longer the case after the Russian Ukrainian war. This sequence of events illustrates how an energy net-exporter can significantly impact the transition trajectory of an energy net-importer, prompting the necessity of maintaining a stable energy net-exporter economy.

Transitioning countries require a nuanced approach that recognizes the unique capabilities and challenges of each energy net-exporter. While there are studies in the literature concerning the transition of net-exporter countries, their availability is limited. For instance, Norway has emerged as a significant player in the journey toward emissions reduction, with recent research noting the dominance of hydropower and wind generation in its power sector, owing to the abundance of these resources in that region.^23^ Given this, a significant increase in electric vehicle (EV) deployment, with penetration rising from 5% in 2014 to 88% in 2022 was observed.^24^ Despite this increase in EVs, Norway’s oil consumption only fell by 11% over the same period,^25^ indicating a complex transition where the adoption of EVs has not equally reduced oil consumption. Furthermore, Norway exports 25% of the EU’s oil and gas demand^26^ and these exports contribute 24% to Norway’s total guanosine diphosphate (GDP),^27^ highlighting the economic dependency on fossil fuels. With carbon targets focused on reducing 90%–95% emissions within Norway’s border by 2050 compared to 1990 levels,^28^ the country faces unique challenges in balancing economic interests and environmental goals. Similarly, Saudi Arabia, the world’s largest oil exporter,^29^ has committed to achieving carbon neutrality by 2060 and was shown to rely on wind generation and imported electricity to sustain the decarbonization targets in the NEOM region.^30^ Despite sharing similar targets and economic infrastructure, the strategies employed by Norway and Saudi Arabia vary as a function of geography and available resources. In Norway, hydropower plays a significant role in its power sector, while in Saudi Arabia, solar and wind energies are pivotal. These contrasting approaches demonstrate that the transition strategies of energy net-exporting countries must be carefully tailored to their specific geographic and resource conditions.

The energy landscape of net-exporter countries is fundamentally driven by their energy conversion and industrial sectors.^31^^,^^32^ While there is limited literature on transitioning these sectors, there remains a significant gap in adopting a wholistic approach, where key sectors are studied in parallel. For instance, Ganzer et al. investigated the transition of oil refining along with other sectors in the UK, revealing early deployment of CCS by 2035 and the offsetting of residual emissions through bioenergy with CCS (BECCS).^33^^,^^34^ Similarly, investigations on how to decarbonize economic sectors in China have emphasized the necessity of integrating negative emissions technologies (NETs), such as BECCS, with other industrial processes.^35^ However, most studies^36^^,^^37^^,^^38^^,^^39^^,^^40^^,^^41^ tend to isolate or only partially examine energy conversion and industrial sectors. As a result, there is a notable lack of research on the transition of key sectors, particularly in the context of energy net-exporter countries, where comprehensive parallel modeling of all sectors remains relatively uncommon.

Given the disproportionate significance of decisions made by net energy-exporting countries to the global energy landscape and associated carbon emissions, quantifying and qualifying societally and technically feasible net-zero transition pathways for these countries is vital. This opportunity is particularly pertinent in the context of the UAE as it strives toward carbon neutrality.^2^ With targets such as increasing oil^42^ and liquefied natural gas (LNG) production by 2030,^43^ questions arise regarding the pathway in which the country can transition without adversely affecting nations reliant on its energy resources. This concern is emphasized by instances in Pakistan, Bangladesh, and Germany, which have reverted to coal when faced with reduced gas supplies.^16^^,^^44^^,^^45^ This becomes especially relevant given the UAE’s focus on diversifying its energy supply domestically, exemplified by recent deployments of nuclear power^46^ and the establishment of one of the world’s largest solar farms.^47^^,^^48^

Considering the aforementioned factors, this study investigates the UAE’s transition toward carbon neutrality. It aims to analyze the power, energy conversion, industrial, and transport sectors concurrently by using the energy systems optimization (ESO) modeling framework. The objective is to pinpoint the specific areas within each sector that should be prioritized for decarbonization in the UAE. Additionally, it seeks to identify the requisite technologies for implementation and takes into account resource availability and energy security. To achieve this, the study addresses the scientific question What are the required capacity developments, technological pathways and associated costs for the UAE to achieve net-zero emissions by 2050 under different resource availability?

Despite the growing body of literature on energy transitions, the majority of existing research has primarily focused on net-importer countries, leaving a significant gap in understanding how major fossil fuel net-exporters can transition to net-zero emissions while maintaining their economic roles. Although some studies have examined decarbonization strategies for net-exporters, they often focus on isolated sectors or specific technologies rather than a comprehensive, system-wide transition.^49^^,^^50^^,^^51^^,^^52^^,^^53^^,^^54^^,^^55^^,^^56^ Furthermore, existing global and regional energy system models^57^^,^^58^^,^^59^ either analyze sectors in isolation, preventing assessment of cross-sectoral impacts or feedbacks, or treat exporting economies such as the UAE, Qatar, Norway, and Denmark as part of aggregated regional nodes rather than as distinct systems. This aggregation overlooks national-scale parameters such as resource endowments, export-sector coupling, and energy-security constraints that critically shape the feasibility and timing of decarbonization. Consequently, these models fail to capture security-constrained choices such as the trade-off between maintaining export output and deploying negative-emission technologies (e.g., BECCS vs. DAC).

This study addresses this research gap by employing a wholistic modeling framework that (i) co-optimizes the power, energy-conversion industries, and transport sectors using hourly profiles aggregated to representative days, (ii) endogenizes export-linked choices (e.g., E-LNG vs. LNG-CCS, refinery retrofits) coupled to power-system decarbonization, and (iii) evaluates negative-emissions options under energy-security constraints (BECCS vs. DAC with biomass import-risk and price sensitivities). By integrating these sectors within a unified optimization framework, this study provides a detailed roadmap for decarbonization that accounts for interdependencies, trade-offs, and sectoral synergies. This research specifically tailors transition pathways to the UAE’s resource constraints, energy security concerns, and policy landscape. This granularity ensures that the study’s findings are not only locally actionable but also globally relevant, offering replicable insights for other fossil fuel net-exporter economies navigating similar challenges.

### Modeling framework

**Figure 1:**
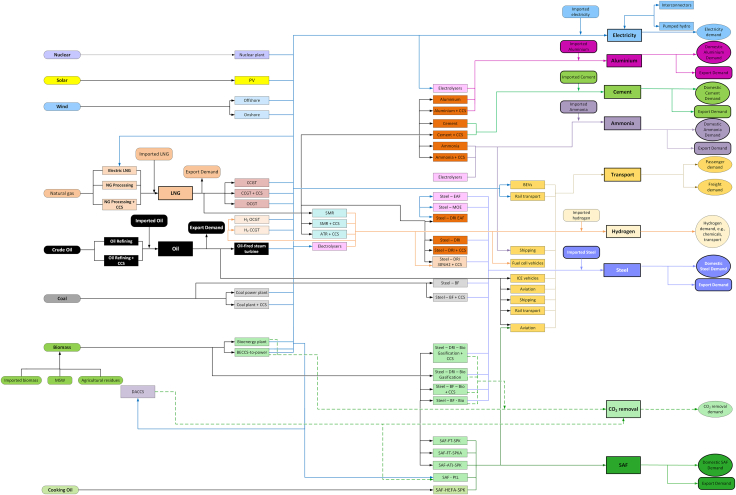
ESO topology illustrating modeled sectors of the UAE

The ESO framework was used to conduct this study, focusing on capacity expansion with unit commitment to meet the demand introduced in the model for the power, energy conversion, industrial,^33^^,^^34^ and transport sectors as shown in Figure 1. ESO is a mixed integer linear programming (MILP) model. It determines the least-cost evolution of these sectors in 5-year time steps by satisfying a range of constraints: hourly power demand, industrial production (including oil, LNG, aluminum, steel, cement, ammonia, and hydrogen) and transport (road transport, aviation, and maritime) sectors. These constraints are supplemented by technical system constraints such as grid reliability and operational parameters, with the requirements being met under a maximum emissions allowance that transitions from current emission levels to net-zero by 2050. Additionally, the model incorporates specific features like technology retrofit and fuel switching. The mathematical formulation of ESO has been presented in previous work,^33^^,^^34^ with details on the transport sector and carbon dioxide removal (CDR) provided in the supplemental information for this publication. Furthermore, the supplemental information outlines the modifications made to ESO to tailor it specifically to the UAE’s unique energy system, policy landscape, and resource constraints (Table S39), ensuring the model accurately reflects the country’s decarbonization challenges and opportunities.

### Scenario definition

As observed in the literature, resource availability plays a critical role in the decarbonization process. To thoroughly examine this impact, a scenario-based approach was employed in the model. This method allows for exploring a range of future conditions to provide a comprehensive understanding of potential outcomes. Given the various pathways available for carbon removal, the scenarios were specifically chosen to assess the influence of permitting nuclear and biomass on the system, particularly on the deployment of technologies and direct air capture (DAC). The following scenarios were implemented in the model:

1. **Nuclear + Biomass:** This scenario combines nuclear expansion with imported biomass from Asian markets like Vietnam and Indonesia, assuming abundant availability. It factors in Asian biomass prices and associated embodied emissions.
2. **Biomass Only:** This scenario utilizes imported biomass from Asian markets such as Vietnam and Indonesia, assuming abundant availability. It considers Asian biomass prices and accounts for associated embodied emissions. Nuclear capacity is capped at the current level of 5.4 GW.
3. **Nuclear + Domestic Biomass:** This scenario permits nuclear expansion and limits biomass use to domestic sources, with availability and prices based locally. Due to the limited available capacity, the size of the BECCS plant is reduced from 500 to 50 MW.
4. **Domestic Biomass Only:** This scenario uses only domestically sourced biomass, with availability and prices based locally. Due to the small available capacity, the size of the BECCS plant is reduced from 500 to 50 MW. Nuclear capacity is capped at the current level of 5.4 GW.

Relevant system parameters are detailed in the supplemental information.

## Results

In December 1971, the distinct Emirates of Abu Dhabi, Dubai, Sharjah, Ajman, Fujairah, Ras Al Khaimah, and Umm Al Quwain unified to form the UAE under the leadership of H.H. Sheikh Zayed bin Sultan Al Nahyan. The UAE is located in the southeastern region of Asia and the eastern part of the Arabian Peninsula, overlooking the Arabian Gulf to the north and northwest, bordered by Saudi Arabia to the west and Oman to the south. Covering 71,023 km^2^ with a 1,318 km coastline, the UAE has a diverse landscape featuring vast desert regions in Abu Dhabi and Dubai, the Hajar Mountains in Sharjah, Fujairah, and Ras Al Khaimah, as well as windy coastal areas along the Arabian Gulf. The country has a population of 9.5 million, experiencing a warm, sunny winter and a hot, humid summer.^60^^,^^61^^,^^62^

Given this unique geographic and climatic context, this section provides a thorough examination of the UAE’s technological strategies aimed at achieving net-zero emissions by 2050 (Figure S2). The analysis below examines the evolution of the power, energy conversion, industrial, and transport sectors. It rigorously evaluates the impacts of the scenarios outlined above on the UAE’s energy infrastructure and CCS adoption. Additionally, it investigates the interdependencies of resource availability, technological deployment and policy implications, thereby offering comprehensive insights for fostering societal and economic benefits through the decarbonization of the UAE.

### Net-zero in the power sector

**Figure 2:**
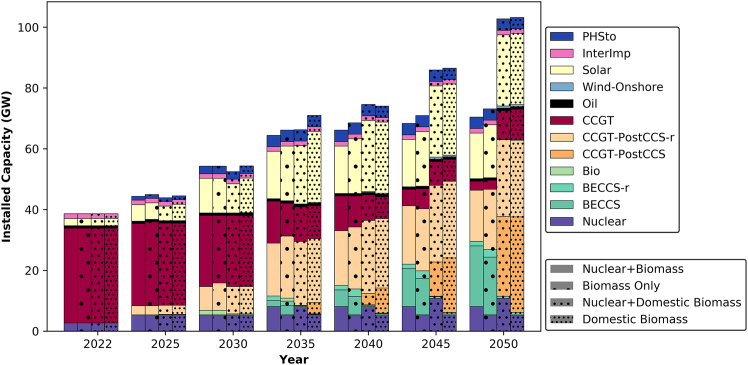
Installed capacity design for different resource scenarios in the UAE Technologies with “-r” at the end represent systems that were retrofitted with CCS at a later stage in their lifetime. Specifically, CCGT-PostCCS-r refers to CCGTs that were initially deployed without CCS and later retrofitted, while BECCS-r represents bioenergy facilities that were later upgraded with CCS.

**Figure 3:**
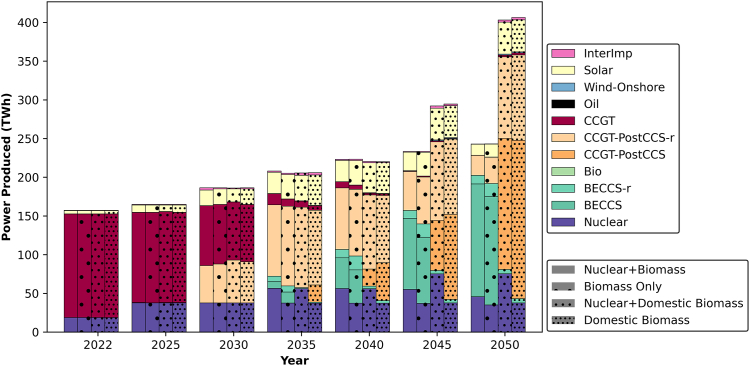
Projected power generation mix in the UAE from 2022 to 2050 Technologies with “-r” at the end represent systems that were retrofitted with CCS at a later stage in their lifetime. Specifically, CCGT-PostCCS-r refers to CCGTs that were initially deployed without CCS and later retrofitted, while BECCS-r represents bioenergy facilities that were later upgraded with CCS.

In analyzing the evolution of the power sector in the UAE, this study considered the availability of resources as a key theme. As shown on the bottom right side of Figures 2 and 3, the shading used on the blocks represents the different scenarios introduced in the model. The capacities are plotted based on these scenarios for each planning period.

The integration of nuclear power into the UAE’s energy mix significantly influences technology deployment. According to the model output, nuclear capacity can reach a maximum of 8 GW in scenarios allowing imported biomass and 11 GW in scenarios utilizing domestic biomass. The availability of nuclear power reduces the system demand for combined cycle gas turbine with CCS (CCGT-CCS) by 3 GW in the imported biomass scenario and by 5 GW in the domestic biomass scenario. Additionally, it increases solar capacity by 2.4 GW in the imported biomass scenario compared to the scenario with domestic biomass. Overall, nuclear power contributes as a firm low-carbon capacity to the grid, adding value to the system by lowering the carbon emissions.

When comparing scenarios using imported vs. domestic biomass, significant variations in total installed capacity are observed. In a domestic biomass scenario, an additional 25 GW of installed capacity is required, primarily to support the DAC capacity (Figure 11) necessary for emissions removal. In the absence of BECCS, the grid relies heavily on natural gas technologies by 2050. Furthermore, an average of an additional 7 GW of solar capacity is needed when domestic biomass is used. A small quantity of onshore wind emerges as a viable option, with a capacity of 700 MW projected in 2050 if imported biomass is not utilized. Of the total installed capacity, the proportion of clean energy averages 67% with imported biomass and 36% with domestic biomass.

It is worth noting that CCS is introduced into the power sector in all scenarios as early as 2025. Moreover, the allowance of imported biomass results in the deployment of a 1.5 GW bio-power plant by 2030, retrofitted with CCS by 2035 and further expanded thereafter. In contrast, when domestic biomass is utilized, limitations in feedstock availability and plant size lead to the deployment of 150 MW of bio-power capacity by 2025, retrofitted with CCS by 2050 to reach a total capacity of 750 MW.

In terms of solar power, the installed capacity is projected to increase by 4–5 times in 2030 compared to 2022 levels. This is exceeding the government’s target of tripling the capacity by 2030.^63^ By 2050, the solar capacity is expected to increase by 10 times compared to 2022 levels in scenarios utilizing domestic biomass. The share of intermittent renewable is projected to stand at 10%–13% in 2050, depending on the scenario.

The projected power generation mix in the UAE, as shown in Figure 3, highlights a diversified approach to meeting growing electricity demand while reaching net-zero by 2050. Rather than a significant reduction in fossil fuel reliance, the results indicate that CCGT with CCS remains a key component of the power system, particularly in scenarios where domestic biomass is used. With increasing electricity demand, power generation expands through a combination of nuclear, solar, BECCS, and abated CCGT. In scenarios where imported biomass is available, BECCS plays a prominent role in power generation, achieving net-negative emissions and minimizing reliance on CCGT-CCS by 2050. However, in scenarios constrained to domestic biomass, the power grid depends more heavily on CCGT-CCS to meet demand. Solar power contributes similarly across all scenarios, complementing nuclear energy in providing low-carbon electricity. While intermittent renewables add to the energy mix, firm power sources such as nuclear, BECCS, and CCGT-CCS remain essential for maintaining grid stability and meeting demand. This balance highlights the trade-offs between biomass availability and the reliance on fossil-based but abated generation technologies.

Overall, the presence of nuclear power in the system favors the deployment of solar capacity, as both technologies have low carbon emissions. However, the feasibility of expanding nuclear capacity in the UAE raises questions due to bureaucratic hurdles^64^ and the substantial increase required (a 2-fold increase compared to current levels in scenarios with domestic biomass). Additionally, while imported biomass facilitates the deployment of BECCS, it raises concerns about energy security, as the availability of imported resources may be unpredictable. If the UAE is to take the imported biomass path and biomass becomes limited during the transition process, the quickest solution would be to use unabated or abated CCGT plants to deliver power. This approach could impact the country’s trajectory toward decarbonization, as no other NETs would be available. Hence, to ensure a smooth transition to carbon neutrality, it is recommended to prioritize the domestic biomass path in the power sector.

### Net-zero in the energy conversion and industrial sector

This section considers the net-zero transition of the energy conversion and industrial sectors in the UAE. The relevance of these scenarios lies in the fact that resource availability governs the decarbonization effort for the production of different commodities, as all sectors are interconnected in the model and impact each other. The four distinct scenarios are only plotted when a variation is caused due to resource availability in the model. Nevertheless, in cases where there are no variations in technology deployment, only a single bar is plotted to visualize the transition.

**Figure 4:**
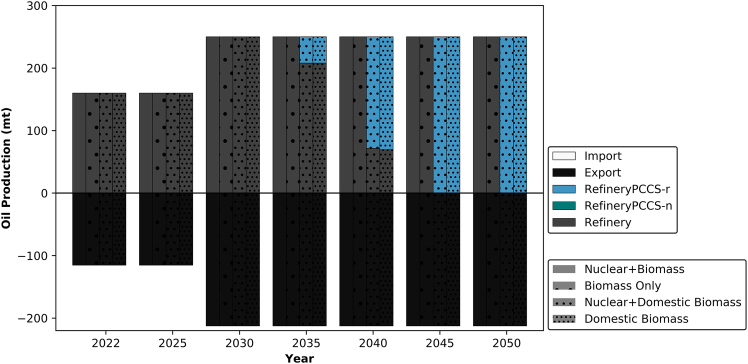
The transition of oil refineries in the UAE RefineryPCCS-r represents a conventional refinery that was initially deployed without CCS and retrofitted with CCS at a later stage of its lifetime.

The output of refineries in the UAE plays a pivotal role in the nation’s economy. Ensuring the stability of resources derived from these refineries is not only crucial for the UAE’s economic security, but also for the countries reliant on the end products. This importance is highlighted by the black bars in Figure 4, which represent the refinery output that is predominantly exported.

Across all examined scenarios in Figure 4, the implementation of CCS for oil refining in the UAE is not deemed essential until 2035, particularly in scenarios utilizing domestic biomass. In these instances, CCS becomes necessary only through retrofitting the capacity. In other words, the capacity developed to accommodate the projected increase in production to 250 million tons per year (Mt/yr)^42^ by 2030 does not necessitate associated CCS facilities until 2035 under domestic biomass scenarios. Conversely, in scenarios utilizing imported biomass, CCS is not required throughout the modeled periods, as BECCS removes the associated emissions from the refining processes.

**Figure 5:**
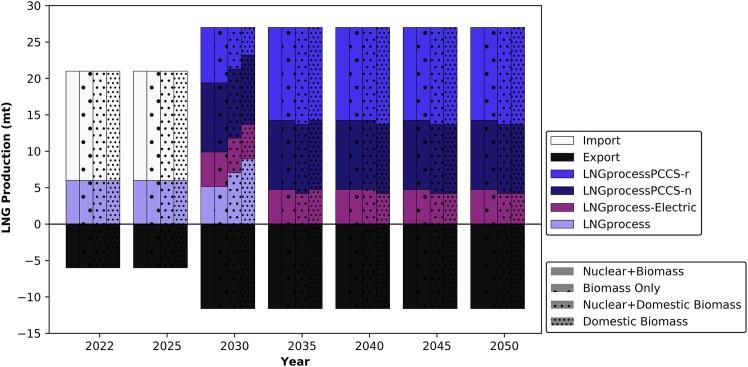
The transition of LNG production in the UAE LNGprocessPCCS-r represents a conventional LNG facility that was initially deployed without CCS and retrofitted with CCS at a later stage of its lifetime. LNGprocess-Electric represents an LNG facility that operates its refrigeration processes using power from the electricity grid.

For LNG, the transition differs from that of oil refineries. As the capacity increases for LNG production in 2030, new technologies emerge. The newly built capacity to produce 27 Mt/yr consists of a blend of technologies. Interestingly, in all scenarios, E-LNG is deployed and it relies on the grid for the refrigeration part of LNG production. The remaining portion of the mix varies depending on the scenario. In terms of existing capacity, all of it is retrofitted with CCS in 2050. The additional share of the 27 million tons (Mt) production target is met by a mix of newly built conventional LNG plants and LNG-CCS plants. Regardless of the scenario, as illustrated in Figure 5, the capacity distribution remains the same in 2050, which consists of a mix of electrically refrigerated LNG and LNG-CCS plants.

**Figure 6:**
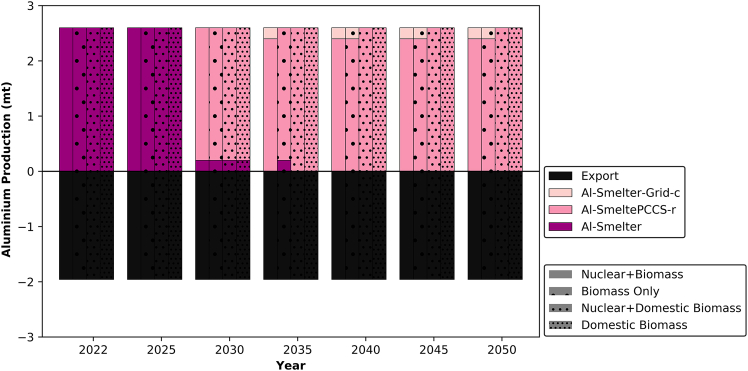
The transition of aluminum production in the UAE Al-SmelterPCCS-r represents a conventional aluminum smelter that was initially deployed without CCS and retrofitted with CCS at a later stage of its lifetime. Al-Smelter-Grid-c represents a conventional aluminum smelter that was initially connected to a CCGT power plant and later transitioned to being connected to the electricity grid.

For aluminum production, known for its high carbon intensity,^65^ understanding the operational dynamics of aluminum smelters in the UAE is crucial before describing the transition process. The energy source for production, which powers the aluminum electrolyzers (Al-smelters), can vary from one country to another.^66^ Typically, an aluminum smelter consists of a CCGT power plant linked to electrolyzers responsible for aluminum production, with a carbon intensity of 5.4 kg-CO_2_/kg-Al.^67^ An important aspect to clarify is the current connection between the existing electrolyzers and the CCGT plants in the model. During the transition process, the model provides the option to either disconnect the electrolyzers from the CCGT plant and connect them directly to the grid or retrofit the existing CCGT plant with CCS. The model includes the three existing smelters in the UAE to produce aluminum. Under all circumstances, the assumed operational factor is 80%.^68^ As shown in Figure 6, two of the plants are retrofitted with CCS by 2030. Beyond that, the fate of the remaining smelter depends on the scenario. In scenarios utilizing imported biomass, this plant is connected to the grid, given that nearly 67% of grid generation is derived from clean energy sources. Conversely, in scenarios with domestic biomass, the remaining plant is retrofitted with CCS.

**Figure 7:**
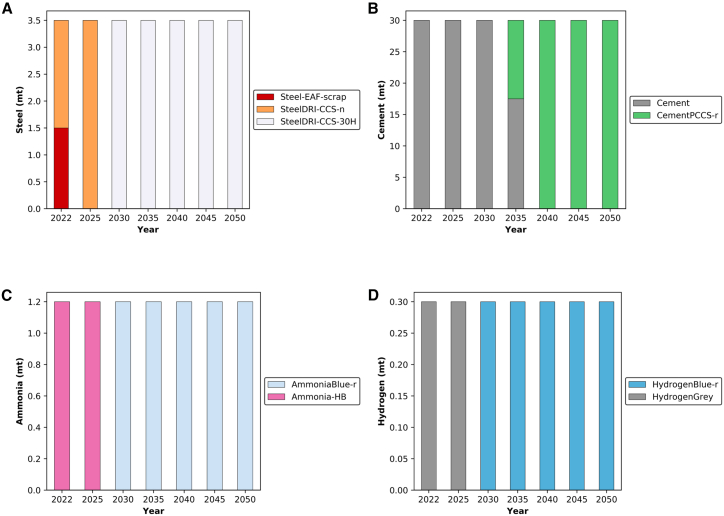
Transition of existing industrial capacities in the UAE from 2022 to 2050 (A) Steel production. (B) Cement production. (C) Ammonia production. (D) Hydrogen production. For steel, SteelDRI-CCS-30H represents the same SteelDRI-n facility that exists in 2025 but operates with a 30% hydrogen blend alongside natural gas. Technologies with “-r” at the end indicate that the technology was initially deployed in its conventional form and retrofitted with CCS at a later stage of its lifetime.

In examining the other commodities, variations in deployment were not observed with respect to resource availability. As shown in Figure 7A, the existing steel production capacity consists of two of the world’s first CCS facilities connected to a steel direct reduced iron (DRI) plant, with the captured CO_2_ utilized for enhanced oil recovery. This collaboration between ADNOC and Emirates Steel involves capturing the CO_2_ in Musaffah area and transferring it via a pipeline to Al Ruwais city.^69^^,^^70^^,^^71^ In terms of transition, Figure 7A illustrates the initial capacity utilizing iron-ore and steel scrap as feedstocks for SteelDRI-CCS and Steel-Electric Arc Furnace (EAF), respectively. Notably, the model suggests replacing Steel-EAF capacity with a new SteelDRI-CCS facility due to the high price of scrap. The price of iron-ore stands at £161/ton,^72^ while scrap costs £322/ton.^73^^,^^74^ However, if scrap prices decrease to £208/ton, Steel-EAF would replace SteelDRI-CCS capacity by 2050. Additionally, the model proposes fuel switching to reduce emissions further and this is by replacing 100% natural gas feedstock with a blend containing 30% hydrogen. Current DRI technology can accommodate this hydrogen blend without process modifications.^75^ This recommendation holds significance not only for the UAE, but also for global steel producers utilizing the SteelDRI process, urging them to consider similar blending levels in their processes.

Unlike other technologies, the transition toward retrofitting with CCS in the cement industry begins to emerge around 2035 as shown in Figure 7B. Notably, over half of the cement capacity is concentrated in the Emirate of Ras Al Khaimah,^76^ located at the far north of the UAE with a coastline overlooking the Arabian Gulf.^77^ Retrofitting with CCS in this area would require further analysis to identify the suitable storage areas. Nevertheless, the assumption of constructing a pipeline to Al Ruwais region is feasible, given that the distance is similar to the existing natural gas transport infrastructure between Qatar, Al Taweela and the Emirate of Fujairah.^78^ Nonetheless, this aspect requires additional research, which falls beyond the scope of this study. Regarding ammonia and hydrogen production, the existing single plants for each are expected to undergo retrofitting by 2030 as shown in Figures 7C and 7D. This aligns with the UAE’s current trajectory, exemplified by the initiation of a pilot plant and the shipment of the first batch of blue ammonia to Germany in 2022.^79^

Based on the findings of this study, insights into the allocation of CCS facilities emerge. Under a domestic biomass scenario, complete retrofitting of oil refining capacity is projected to be necessary by 2045. For LNG, CCS implementation occurs more rapidly due to the plant’s higher carbon intensity in comparison to oil refining. It is noteworthy that a significant portion of the refining capacity and natural gas processing facilities are situated in Al Ruwais industrial city in the UAE, which is in close proximity to depleted fossil wells that are suitable for CO_2_ storage. Regarding other commodities, all except cement will require retrofitting by 2030 due to their higher carbon intensity in producing 1 Mt compared to other commodities as shown in Table 1.

**Table 1:** Carbon intensity for producing 1 metric ton of each commodity using different technologies

Technology^33^^,^^67^^,^^80^^,^^81^^,^^82^	Carbon intensity (Mt-CO_2_/Mt-X)	Emissions source
Al-Smelter	5.4	processing and CCGT plant
Cement	0.6	clinker and reactors
Ammonia-HB	2.4	SMR furnace and reactors
Hydrogen grey	9.3	SMR furnace and reactors

### Net-zero in the transport sector

**Figure 8:**
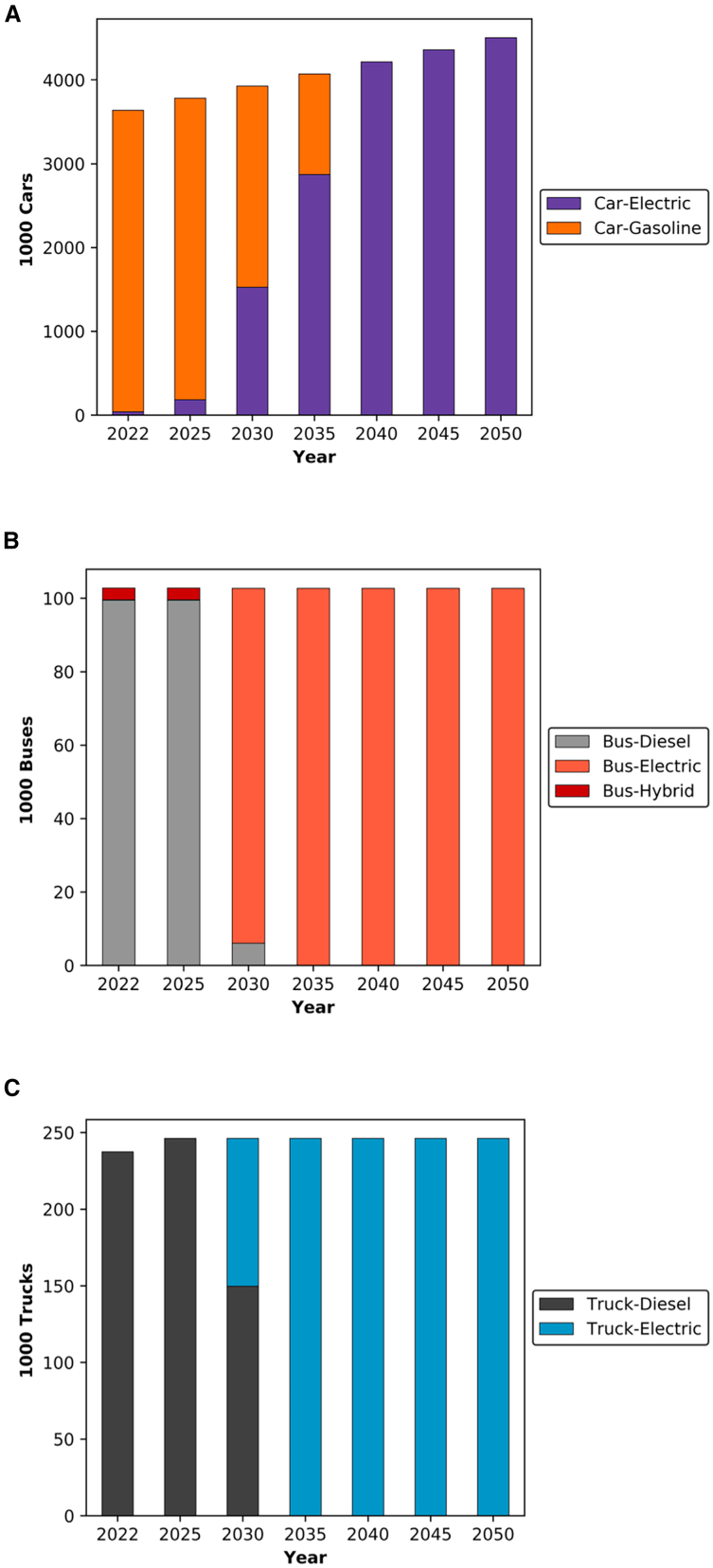
Transition of road vehicles in the UAE (A) The transition of individual car vehicles in the UAE. (B) The transition of mini buses in the UAE. (C) The transition of heavy-duty vehicles in the UAE.

The output of the transport sector remained consistent across the four scenarios analyzed. Specifically, this study focused on road vehicles, sea, and air transport. Examining road vehicles in particular, Figure 8 illustrates a notable trend that is the electrification of all road vehicles by 2050. This shift is primarily attributed to the assumed price of oil, with EVs emerging as a more cost-effective option compared to internal combustion engine (ICE) vehicles. Relative to the UAE’s latest targets aiming for EV adoption of 13.5% in 2030, this analysis suggests a more accelerated approach may be needed to realize the economic benefits of decarbonizing the transport sector. Notably, the targets published by the UAE’s Ministry of Climate Change and Environment (MOCCAE) in the UAE indicate ambitions of reaching 53% EV penetration for cars and 60% for buses by 2050.^83^

By examining the targets set by the MOCCAE, the analysis indicated that failing to achieve 100% electrification by 2050 would incur an additional cumulative cost of £25 billion in scenarios involving domestic biomass. This cost covers the deployment of the additional DAC along its associated power requirements and fuel costs for operating ICE vehicles. Considering potential oil price decreases, it is important to note that in most countries, natural gas prices are linked to market oil prices in long-term contracts.^84^ Consequently, during oil price fluctuations, transitioning to EVs will remain economically favorable as both fuel prices are expected to move together, given that the grid in the UAE will be predominantly powered by natural gas.

This highlights the importance for policymakers to prioritize robust electrification efforts within the transport sector. Regarding vehicle availability to support such a transition, the UAE has previously made significant strides in this direction with plans to establish its first EV manufacturing plant.^85^ Furthermore, the UAE hosts one of the largest used car markets globally, valued at $20 billion in 2021.^86^ Thus, constraints on vehicle availability are unlikely. Moreover, in terms of replacing the existing capacity, vehicle licenses are typically not renewed after 15 years of operation in the UAE.^87^

**Figure 9:**
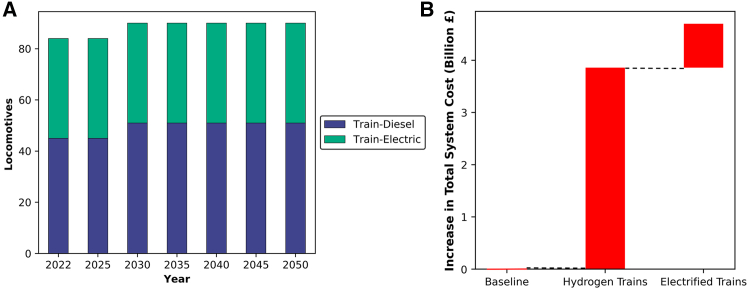
Projected transformation of UAE railway locomotives and associated system costs under alternative technology pathways (A) Projected composition of train locomotives in the UAE from 2022 to 2050, showing the distribution between diesel and electric trains. (B) Increase in total system cost (£billion) associated with transitioning to hydrogen-powered or electrified trains compared to the baseline scenario.

For trains, the current electric capacity represents the existing trains in the Emirate of Dubai, as shown in Figure 9A. This capacity in the system is dedicated for passenger transport within the city (Emirate) itself and remains as is until 2050 without favoring a switch to diesel trains due to the travel distance. For transportation between the different Emirates of the UAE, the system tends to opt for diesel trains for both passenger and freight transport. This preference arises from the substantial electricity demand that would be placed on the grid by electrified trains, especially considering the long distances traveled. Consequently, given the relatively low cost of natural gas used to power DAC, the model indicates that operating these trains using diesel fuel is the more economical option. However, transitioning to hydrogen-powered trains would increase the total system cost by £4 billion, as illustrated in Figure 9B. Electrifying train transport would require an additional 2 GW of power capacity to support both train operations and the increased DAC demand. This shift would result in an estimated £5 billion rise in the total system cost.

**Figure 10:**
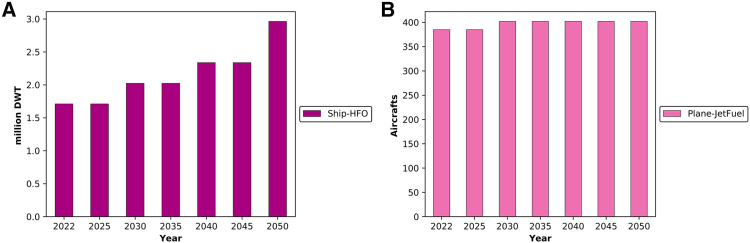
The transition of sea and air transport in the UAE (A) Maritime transport expressed as total ship deadweight tonnage. (B) Air transport capacity expressed as the number of aircraft.

In the UAE’s latest Nationally Determined Contributions (NDCs), sea and air transport were not accounted for.^83^ In contrast, in this study, these critical components of the transport sector were incorporated to comprehensively assess the total emissions as shown in Figure 10. The inclusion of sea and air transport in the model allowed for a more holistic analysis of emissions across sectors. It is noteworthy that the fuel used in these modes of transportation in 2050 consists of low-carbon fuels. In this part of the analysis, the age of each plane and ship was taken into account, along with the associated costs of replacing the old capacity and the deployment of additional targeted capacities.^88^^,^^89^^,^^90^^,^^91^

### Sectoral emissions and total system cost

**Figure 11:**
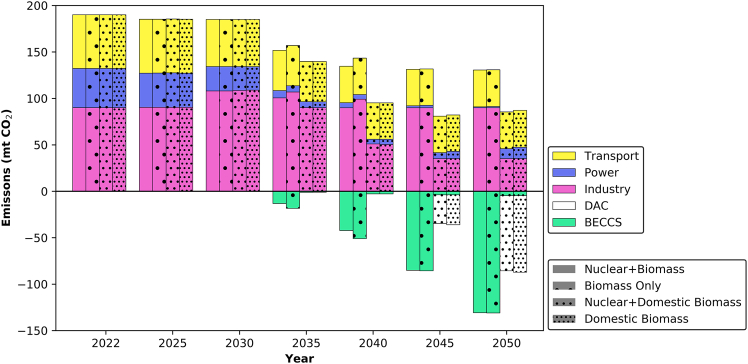
The transition of sectoral emissions in the UAE

The preceding sections have comprehensively addressed the transition within each modeled sector. Analyzing the system emissions revealed that the energy conversion and industrial sectors emerges as the most carbon-intensive sector among the modeled sectors, as shown in Figure 11. Accordingly, the transition within the model initiates with the decarbonization of the power sector, as previously demonstrated, wherein CCGTs are partially retrofitted with CCS as early as 2025. Transition efforts within the energy conversion and industrial sector commence in 2030, coinciding with the increased production of oil and LNG. In this context, CCS plays a dominant role, particularly in the power sector, thereby facilitating the electrification of LNG refrigeration and road vehicles.

In scenarios where BECCS is deployed to remove emissions, higher emissions from the energy conversion and industrial sector are observed primarily due to not abating the oil refineries. This is because BECCS can remove emissions while simultaneously providing power, making it more economically advantageous to allocate CCS capacity to. In scenarios utilizing domestic biomass, DAC predominantly offsets carbon emissions due to limitations in domestic biomass availability. The residual emissions observed in domestic biomass scenarios in Figure 11 represent emissions that cannot be captured due to technology constraints. Based on this, the DAC requirement in the UAE is projected to be around 84 Mt-CO_2_ by 2050. The carbon intensity of the power sector under domestic biomass scenarios is estimated to be at 0.03 t-CO_2_/MWh in 2050, with the 2030 level reaching 0.14 t-CO_2_/MWh, which is lower than the published NDC value of 0.27 t-CO_2_/MWh.^83^

It is worth noting that depending on future collaborations, there may be a possibility of a higher share of BECCS in the system if imported biomass is integrated. Should this resource become available, pursuing this path holds potential, as it is more cost-effective for removing carbon while contributing to the grid demand and its requirements.

**Figure 12:**
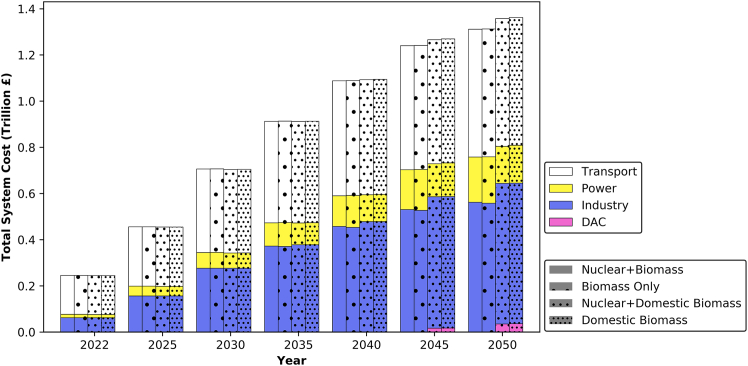
Sectoral cumulative total system cost under the four scenarios, including investments needed to meet the increase in power demand, expansion of oil production to 250 Mt/yr^42^ and LNG to 27 Mt/yr,^43^ as well as investments for expanding sea and air transport and replacing old capacities in the transport sector

Upon examining the cost implications of the four scenarios, a slight variance in the cumulative total system cost is observed as shown in Figure 12. This is with the domestic biomass scenarios proving to be £48 billion more expensive. In a hypothetical scenario where BECCS is employed to remove emissions, the required biomass in 2050 would amount to 88 Mt. The substantial biomass requirement raises concerns regarding energy security, particularly as the UAE lacks local biomass resources. Moreover, relying on international resources via sea routes poses risks of disruption, highlighting the importance of prioritizing domestic biomass for energy security. Implementing DAC alongside domestic biomass ensures the UAE’s trajectory toward achieving net-zero emissions by 2050. The additional £48 billion investment is therefore deemed necessary to secure net-zero by 2050 in the UAE.

**Figure 13:**
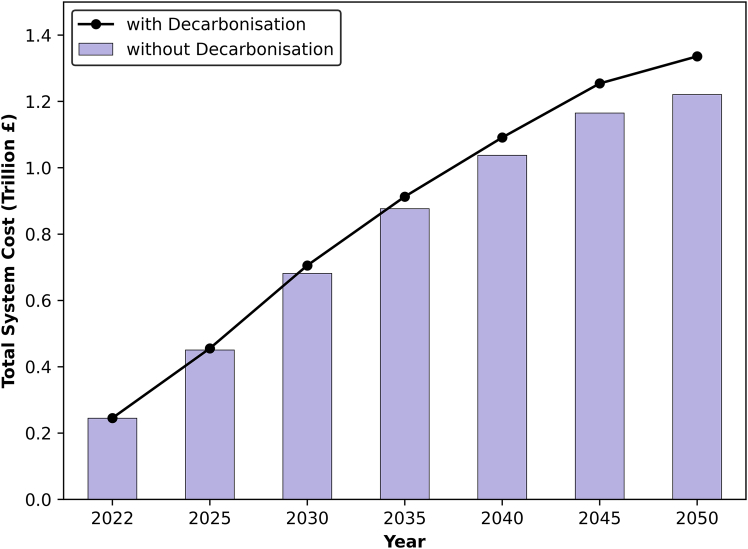
Cumulative total system cost comparison between BAU (purple bars) and decabronization scenarios (line plot) Costs include investments needed to meet the increase in power demand, expansion of oil production to 250 Mt/yr,^42^ LNG production to 27 Mt/yr,^43^ as well as investments for expanding sea transport, air transport, and replacing old capacities in the transport sector.

By adhering to future targets, Figure 13 illustrates two contrasting scenarios: a business as usual (BAU) approach, which represents the UAE maintaining its current practices without implementing carbon mitigation measures and capping emissions at today’s level, contrasting with a decarbonization scenario. Both scenarios consider the expansion of oil and LNG production, alongside investments needed to meet the increase in power demand, the expansion of sea transport, air transport and the replacement of old capacities in the transport sector. The decarbonization cost shown in the figure represents the average cost across the four scenarios. It is evident from the figure that the difference in cost between these two scenarios amounts to £115 billion, which is 9.4% higher than BAU. Therefore, in order to achieve carbon neutrality, the UAE would need to allocate approximately £4 billion annually for investments in decarbonization efforts, that is equivalent to 1.3% of its annual gross value added (GVA).^92^

### Sensitivity

The previous results provide a comprehensive overview of the transition process in the UAE to net-zero by 2050. In the following section, an examination of key technologies pivotal to decarbonizing the UAE economy is further analyzed. The technologies selected for this section are DAC, BECCS, and Solar.

As previously discussed, the UAE’s economy relies heavily on energy conversion and industrial activities, which must be maintained during the net-zero transition to ensure economic stability. In this context, this section evaluates the performance of NETs in the model, focusing on DAC Capital Expenditure (CAPEX) and biomass pricing. DAC CAPEX is a key consideration due to the novelty of the technology. In this study, the DAC CAPEX corresponds to the n-th produced technology, with a levelized cost averaging around $476 per ton of captured CO_2_.^93^ The assessment of DAC CAPEX aims to identify when DAC becomes competitive with BECCS, which has been consistently favored in imported biomass scenarios. This analysis was conducted under Only Biomass scenario.

**Figure 14:**
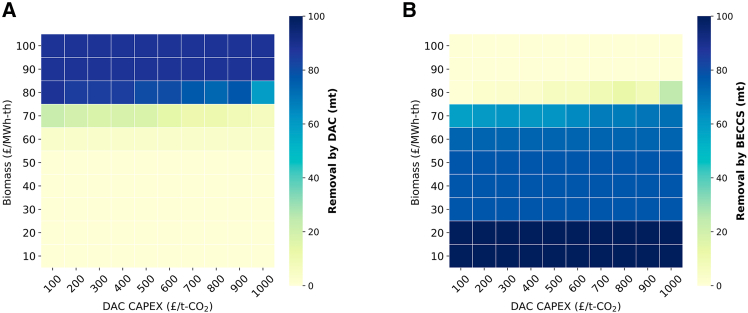
Comparison of carbon removal strategies for 2050 (A) The impact of biomass price and DAC CAPEX on carbon removal via DAC. (B) The impact of biomass price and DAC CAPEX on carbon removal via BECCS.

Analysis shown in Figure 14 suggests that the deployment of DAC is largely unaffected by its CAPEX. Instead, it is more influenced by the biomass price. In a scenario where market forces drive biomass prices four times higher than its current levels, DAC deployment increases significantly, regardless of its CAPEX.

The sensitivity results of BECCS vs. DAC showcase the importance of investing in research aimed at cultivating environmentally friendly biomass within the UAE at prices below £70/MWh_th_. Such investments would not only enhance the country’s energy security, but also facilitate the widespread adoption of BECCS technology in the UAE. This would enable larger-scale power plants and more effective carbon removal.

In the context of domestic biomass scenarios, the deployment of solar energy in the UAE is constrained by the build rate limitations integrated in the model. This build rate is benchmarked against the world’s largest solar farm (Noor) that is situated in Abu Dhabi.^48^

**Figure 15:**
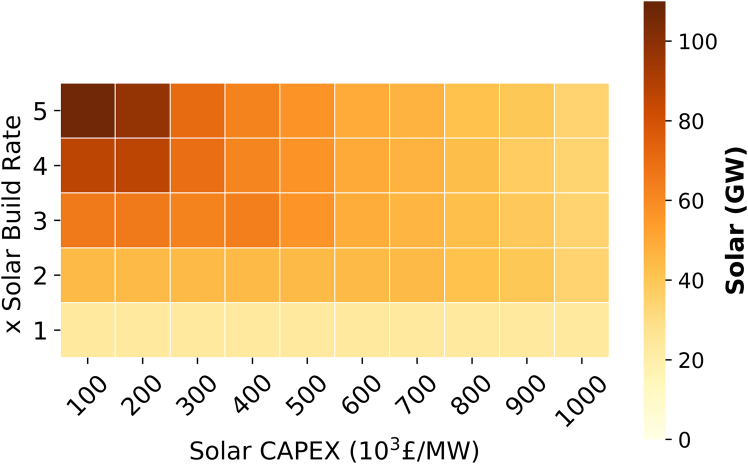
The impact of solar build rate and CAPEX on solar installed capacity in 2050

To evaluate the required solar capacity in a decarbonized UAE, a sensitivity analysis was conducted by varying the build rate of solar farms against their CAPEX as illustrated in Figure 15. In all the solar scenarios examined, the capacity of natural gas technologies remained consistent, as per the grid requirement for maintaining reserve (20% back-up^94^) and inertial rotating capacities. As a result, the substitution of natural gas with solar energy was only in power generation. The solar CAPEX utilized in the model approximates around £616 per kW, and this is based on current investments in solar farms within the UAE.^95^ Analysis of varying CAPEX levels revealed a positive correlation between decreasing prices and increasing build rates for solar infrastructure. Nevertheless, irrespective of CAPEX and at 2x build rate, the deployed solar remained at a 40 GW capacity. This highlights the crucial requirement for the UAE to double its historical build rate to achieve a capacity of 40 GW by 2050 in comparison to the 23.4 GW under the domestic biomass scenario. This expansion would necessitate an area of 156 km^2^ from the current 16 km^2^^47^ that is equivalent to 139 times the land size of Dubai Mall.^96^

Notably, despite the significant solar expansion, the reduction in total system costs was marginal with savings of less than 1% at a CAPEX of £100k per kW. Therefore, the justification for such extensive solar deployment hinges on potential reductions in reserve capacity requirements or the availability of commercial-scale storage solutions.

## Discussion

Despite the increasing global focus on carbon neutrality, there has been a limited understanding of how energy net-exporter countries can transition toward this goal while maintaining their pivotal role in global energy supply. Here, the necessity of studying the transition pathways of such countries, which often differ significantly from those of net-importers, is demonstrated. One key lesson from existing transitions, such as Norway’s high EV penetration without a corresponding significant drop in oil consumption, is that single-sector transitions may not always lead to substantial reductions in fossil fuel demand unless accompanied by broader systemic changes. This study highlights the importance of a holistic approach, where sectoral transitions are analyzed in parallel, ensuring that decarbonization in one sector is reinforced by complementary efforts in others. By examining the UAE’s transition toward carbon neutrality across its key sectors, insights into the unique challenges and opportunities faced by energy net-exporters are provided.

**Figure 16:**
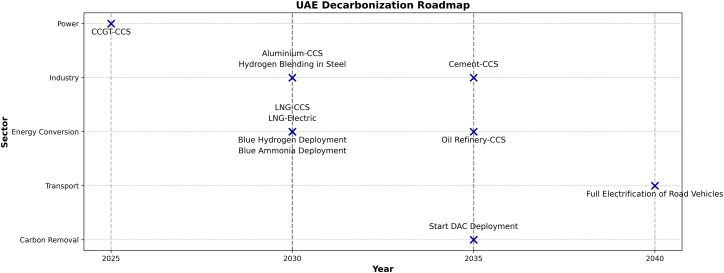
UAE decarbonization roadmap outlining the planned deployment of key decarbonization technologies across sectors

Although global outlooks^97^^,^^98^ provide useful insights into how policy pledges influence energy transitions, they do not determine technology choices through cross-sectoral optimization at the national level. This study addresses that gap by identifying sector-specific technology pathways derived from a least-cost, system-wide optimization for the UAE. Unlike descriptive timelines in global outlooks, Figure 16 is a synthesis of the optimization results that translates least-cost, cross-sectoral deployment sequences into a decarbonization roadmap. Rather than projecting generic technology uptake, it quantifies when and why each technology becomes cost-optimal under the UAE’s security and export constraints as explained under the results section. The findings highlight the sectoral timeline of decarbonization efforts and the critical role of CCS deployment in mitigating carbon emissions across key industries. As illustrated in Figure 16, the power sector is projected to implement CCGT-CCS as early as 2025, emphasizing the urgency of integrating CCS technologies into fossil-based power generation. This early action sets the stage for deeper decarbonization across other sectors in the following decade.

In the energy conversion sector, CCS deployment in LNG processing is anticipated by 2030, alongside the diversification of LNG facilities to include LNG-electric plants. This will further strengthen the UAE’s commitment to reducing emissions from hydrocarbon exports, demonstrating a viable pathway for lowering the carbon intensity of LNG. By 2035, the complete retrofitting of oil refining capacity with CCS will play a crucial role in enhancing cleaner refining processes, significantly contributing to the sector’s decarbonization efforts. The industrial sector also plays a key role in this roadmap, with aluminum CCS deployment and hydrogen blending in steel expected by 2030, followed by CCS retrofits in cement by 2035. These milestones underscore the growing importance of hydrogen and CCS integration in hard-to-abate industries, where direct electrification remains challenging.

In carbon removal efforts, DAC deployment is expected to begin around 2035, signaling the UAE’s long-term reliance on NETs. Meanwhile, the road transport sector is projected to reach full electrification by 2040, reinforcing the importance of prioritizing transport electrification due to its substantial economic and emissions reduction benefits.

The roadmap also highlights the importance of biomass integration, particularly if supplemented with imported sources. The increased availability of BECCS presents a cost-effective opportunity for achieving negative emissions while supporting grid stability. However, this study emphasizes the need for further research into the sustainability and cost-effectiveness of biomass cultivation, particularly under domestic resource constraints. Ensuring environmentally responsible biomass utilization below specified cost thresholds will be crucial in maintaining energy security while advancing decarbonization objectives.

Overall, these findings provide valuable insights for policymakers, industry stakeholders, and researchers, emphasizing the need for collaborative efforts to scale CCS deployment, enhance electrification strategies and integrate sustainable biomass solutions into the UAE’s energy transition. Further research is recommended to assess the role of alternative fuel utilization, the socioeconomic implications of decarbonization, and potential policy mechanisms to ensure a smooth and sustainable transition toward carbon neutrality.

### Limitations of the study

This study assumes that energy demand remains stable despite potential global policy shifts. While stringent climate policies may impact fossil fuel demand, long-term contracts between the UAE and key energy importers (e.g., Germany, China, and Japan^99^^,^^100^) provide a degree of demand stability. However, future global energy market uncertainties may necessitate further revisions to this assumption.

## Resource availability

### Lead contact

Further information and requests for resources should be directed to and will be fulfilled by the lead contact, Niall Mac Dowell (niall@imperial.ac.uk).

### Materials availability

This study did not generate new unique reagents.

### Data and code availability

• All datasets generated and analyzed during this study are provided in the supplemental information. No external repository accession numbers were generated.
• The ESO model is available at zenodo.^101^ The new additions and modifications applied to the original ESO code are included in the supplemental information.
• Any additional information required to reanalyze the data reported in this paper is available from the lead contact upon request.

## Acknowledgments

This work was completed without external funding or specific acknowledgments.

## Author contributions

Conceptualization, N.M.D.; investigation, R.Y. and N.M.D.; writing – original draft, R.Y.; writing – review & editing, R.Y. and N.M.D.; funding acquisition, R.Y.; supervision, N.M.D.

## Declaration of interests

The authors declare no competing interests.

## STAR★Methods

### Key resources table

**Table:** 

REAGENT or RESOURCE	SOURCE	IDENTIFIER
**Deposited data**		

Availability of renewable energy sources	Renewables Ninja	https://www.renewables.ninja/
Model inputs	This paper (supplemental information)	S1

**Software and algorithms**		

Energy Systems Optimization model	Imperial College London	https://doi.org/10.5281/zenodo.1048943
UAE energy system model	This paper (supplemental information)	S1

### Method details

#### Framework definition

In recent research focusing on the ESO-X model, Ganzer^33^ brought attention to a significant gap in numerous energy models, particularly the lack of consideration for the industrial sector. Responding to this gap, an extension of the ESO-X model was developed to incorporate the industrial sector and its social economic impact. Building upon this foundation, the present study explores the representation of the transport sector and the addition of DAC within energy models. The adaptation of the model to the UAE is elaborated upon in the supplemental information where Figure S3 illusrates the emissions trajectory adopted in the model.

#### The power sector module

In detailing the power sector within the ESO-X model, comprehensive formulations are provided in publications by Ganzer^33^ and Ganzer et al.^34^ Here, an overview will be presented, focusing on key aspects of the model pertaining to the power sector. The most relevant segment of the model to this work is addressing system requirements. This segment encompasses the electricity supplied by the grid and storage technologies to meet the energy requirement of hourly system demand and transmission losses while considering unmet demand. This component of the model has undergone further modifications to incorporate the electrical demand of the industrial and transport sectors. The final adjustment to this formula is presented in the supplemental information document. Moreover, the model integrates constraints related to the gird such as system inertia and reserve margin. For these constraints, a constant value is assumed irrespective of the system design.

To effectively represent the power sector, the model accounts for the retrofitting of existing unabated fossil capacity with carbon capture and storage (CCS). This strategic inclusion is designed to prevent premature decommissioning of new plants in alignment with the planned net-zero targets. Beyond this, the model incorporates the option of early decommissioning for technologies when retrofitting or maintaining the existing technology is not economically viable. This choice is particularly relevant due to the model’s consideration of no-load Operational Expenditure (OPEXNL). The model reflects this by considering existing coal, Combined Cycle Gas Turbine (CCGT) and biomass power plants as candidates for retrofitting with CCS.

It is important to highlight that a time clustering approach was employed to profile the hourly data input for electricity demand along solar, onshore and offshore wind availability profiles. This approach facilitates the computational efficiency of the model using the k-means clustering algorithm approach. The k-means algorithm is an iterative method that partitions datasets into subgroups, ensuring each data point belongs exclusively to one group. These data points are then assigned to a cluster in a manner that minimises the sum of squared distances between the data point and the cluster’s centroid. To maintain consistency, all data are normalised by the maximum value present in each dataset. The outcome of this pre-processing step is the formation of 11-day clusters from the full-year hourly profiles, with each day encompassing 24 hours. This method enhances the model’s ability to handle temporal variations in electricity demand and intermittent renewable energy availability efficiently as shown in Figure S2.

Tables 1, S5, and S6 describe the technologies in this segment of the model.

#### The industrial sector module

In the industrial section of the model, the production of industrial products is addressed following the mathematical formulation outlined in the work of Ganzer.^33^ This segment considers the emissions associated with the production process to meet domestic demand while accounting for both import and export. A noteworthy novel extension to this model segment involves the inclusion of various products, each associated with its distinct production technologies. The added products are liquefied natural gas (LNG), aluminium, ammonia, hydrogen and SAF. The corresponding technologies are detailed in Tables S2 and S7–S29. This addition enhances the model’s capability to comprehensively capture the emissions and technological intricacies with the power sector for industrial production.

#### The transport sector module

To incorporate the transport sector into the model, the formulation shown in the supplemental information were integrated in the ESO-X model with the nomenclature detailed in Table S4. This comprehensive representation of the transport sector in the model ensures a detailed consideration of various modes (road, sea and air), technologies and associated emissions. The technologies used to meet the demand for each mode of transportation are outlined in Tables S3 and S30–S38.

The Technologies introduced to the ESO-X are highlighted below:

• Conventional fossil fuel generation: Combined-cycle gas turbine (CCGT) and oil-fired power plants (Oil).
• Interconnection with external electricity markets is modeled (InterImp). It is assumed that no further electrical interconnections will be built with Kingdom of Saudi Arabia (KSA) due to the frequency mismatch (KSA operates at 60 Hz,^102^ while the UAE operates at 50 Hz^103^). Similarly, it is assumed that no additional capacity will be built with Oman, as the existing capacity is considered sufficient.
• Firm low-carbon generation: Nuclear, biomass (bio) and CCGT plants equipped with post-combustion CCS (CCGT-CCS), which can be retrofitted to existing technologies (CCS-r).
• Intermittent low-carbon generation: Solar photovoltaics (PV), onshore and offshore wind turbines.
• Energy storage: Pumped hydroelectric storage (PHSto).
• Oil production: Conventional refinery (Refinery), refinery with CCS (RefineryPCCS-n) and existing refinery retrofitted with CCS (RefineryPCCS-r).
• LNG production: Conventional natural gas processing plant with refrigeration for LNG production (LNGprocess), LNG plant with CCS or CCS retrofit (LNGprocessPCCS or LNGprocessPCCS-r) and LNG plant using electric refrigeration (LNGprocess-Electric).
• Aluminium smelter: Aluminium electrolyzers connected to a CCGT power plant (Al-Smelter), with options for building with CCS, retrofitting existing facilities with CCS (Al-Smelter-CCS, Al-Smelter-CCS-r) or connecting existing facilities to the grid (Al-Smelter-Grid-c).
• Ammonia production: Haber Bosch process (Ammonia-HB), which can be retrofitted with CCS (AmmoniaBlue-r) and green ammonia production.
• Hydrogen production: Steam reformer (HydrogenGrey) and options for retrofitting with CCS (HydrogenBlue-r) and green hydrogen production.
• Road transport: Internal Combustion Engine (ICE) and electric vehicles specific to the UAE that include Car-Gasoline, Car-Electric, Bus-Diesel, Bus-Electric, Truck-Diesel and Truck-Electric.
• Sea and air transport: Vessels using heavy fuel oil (Ship HFO) and planes running on jet fuels (Plane-JetFuel).
• Carbon dioxide removal (CDR): Bioenergy with carbon capture and storage (BECCS) contributing to power generation and direct air capture systems (DAC) with power requirements were used for carbon dioxide (CO_2_) removal.

### Quantification and statistical analysis

This study is based on an MILP energy system optimisation model. All quantitative results shown in the main text are direct outputs of the optimisation model for each scenario described under “scenario definition” in the modeling framework.

All optimisation problems were implemented in GAMS and solved using the CPLEX solver. Further implementation details, including model formulation and scenario definitions, are provided in the supplemental information.
