# Supplementary material for: Decarbonizing the emirates: A roadmap to net-zero emissions by 2050 in the UAE
Source: iScience. 2025 Dec 8;29(1):114348. doi: 10.1016/j.isci.2025.114348 (PMC12799796; doi:10.1016/j.isci.2025.114348)
Supplement: Document S1. Figures S1–S3 and Tables S1–S39 [file mmc1.pdf]

iScience, Volume *29*

Supplemental information

Decarbonizing the emirates: A roadmap to net-zero emissions by 2050 in the UAE

Roghayeh Yousef and Niall Mac Dowell

Supplementary Information

List of Acronyms

**BECCS**	Bioenergy with Carbon Capture and Storage
**bio**	Biomass-Based
**CAPEX**	Capital Expenditure
**CCGT**	Combined Cycle Gas Turbine
**CCS**	Carbon Capture and Storage
**CCUS**	Carbon Capture, Utilisation and Storage
**CDR**	Carbon Dioxide Removal
**CO_2**	Carbon Dioxide
**DAC**	Direct Air Capture
**DRI**	Direct Reduced Iron
**EAF**	Electric Arc Furnace
**ESO**	Energy Systems Optimization
**EU**	European Union
**GCC**	Gulf Cooperation Council
**GDP**	Gross Domestic Product
**GHG**	Greenhouse Gas
**GVA**	Gross Value Added
**H.H.**	His Highness
**HEFA**	Hydroprocessed Esters and Fatty Acids
**HFO**	Heavy Fuel Oil
**Hz**	Hertz (Frequency)
**IAM**	Integrated Assessment Model
**ICAO**	International Civil Aviation Organisation
**ICE**	Internal Combustion Engine
**IRENA**	International Renewable Energy Agency
**JEDI**	Job and Economic Development Impact
**KSA**	Kingdom of Saudi Arabia
**LNG**	Liquified Natural Gas

**MENA**	Middle East and North Africa
**MILP**	Mixed-Integer Linear Programming
**MSW**	Municipal Solid Waste
**mt**	million ton(s)
**mt/yr**	million ton(s) per annum
**NDC**	Nationally Determined Contributions
**NETs**	Negative Emissions Technologies
**OPEX**	Operating Expenditure
**OPEXFix**	Fixed Operating Expenditure
**OPEXNL**	Non-Linear Operating Expenditure
**OPEXSU**	Operating Expenditure for Start-Up
**PHSto**	Power-to-Hydrogen Storage
**PtL**	Power to Liquid
**SAF**	Sustainable Aviation Fuel
**UAE**	United Arab Emirates
**UK**	United Kingdom
**UN**	United Nations
**UNFCCC**	United Nations Framework Convention on Climate Change
**US**	United States

Framework

Energy Systems Optimisation (ESO) Framework

Mainly, the ESO version is employed when detailed technical operation is required [S1], where the ESO-X version focuses on capacity expansion. ESO-XEL examines the effect of a technology learning system [S2] and ESONE is utilised when a focus on the spatio-temporal distribution of energy transition is necessary [S3]. As the focus of this work is to achieve cost-optimal transition through capacity expansion, perfect foresight is assumed to be the most suitable approach. Accordingly, the ESO-X version is selected to model the energy systems of both the UK and the UAE. This model is a Mixed-Integer Linear Programming (MILP) model designed to minimise the total system cost. It operates on a multiscale framework, incorporating capacity expansion with unit commitment and utilising a 5-year planning period. Furthermore, this model utilises the S curve approach, which facilitates the growth of emerging technologies beyond historical build rates.

Table S1: List of technologies used in the power sector segment of the ESO model [S2].

Technology	Description
Nuclear	A facility that generates electricity through controlled nuclear reactions.
Coal	A steam turbine facility that produces electricity by burning coal, which heats water in a boiler to produce steam that drives a turbine connected to a generator.
Bio	A steam turbine facility that generates electricity using biomass, such as wood chips or agricultural waste.
CCGT	A combined cycle gas turbine facility that produces electricity by burning natural gas in a gas turbine, with the waste heat used to generate additional power in a steam turbine.

Continued on next page

Table 1 – continued from previous page

Technology	Description
OCGT	A facility that generates electricity using an open cycle gas turbine, which burns natural gas to drive a generator directly.
Coal-PostCCS	A new coal-fired power plant equipped with CCS technology.
Coal-PostCCSr	An existing coal-fired power plant retrofitted with CCS technology.
CCGT-PostCCS	A new natural gas-fired power plant equipped with CCS technology.
CCGT-PostCCSr	An existing natural gas-fired power plant retrofitted with CCS technology.
BECCS	A facility that generates electricity using biomass and incorporates CCS technology to achieve negative emissions by capturing and storing CO_2.
BECCSr	An existing facility that generates electricity using biomass that is retrofitted with CCS technology to achieve negative emissions.
Wind-Onshore	A wind farm located on land that generates electricity using wind turbines.
Wind-Offshore	A wind farm situated offshore that generates electricity using wind turbines.
Solar	A facility that generates electricity using solar energy through photovoltaic panels.
InterImp	A cross-border high voltage direct/alternative current electricity interconnector that facilitates the import of electricity from neighboring countries.
PHSto	Pumped hydro storage technology, involving two reservoirs at different elevations, where water is pumped to the upper reservoir during periods of low electricity demand and released to the lower reservoir during high demand to generate electricity.
Oil	A facility that generates electricity using a diesel generator fueled by diesel oil.
H_2-CCGT	A facility that generates electricity using a combined cycle gas turbine fueled by hydrogen .

Table S2: List of technologies used in the industrial sector segment of the ESO model [S4, 5, 6, 7, 8, 9, 10, 11].

Technology	Description
Cement	A facility specialising in cement production utilising kiln technology.
Cement-PCCS-r	An existing cement production facility retrofitted with CCS technology.
Cement-PCC-n	A newly constructed cement production facility equipped with CCS.
Cement-Oxy-r	A cement production plant retrofitted with oxy-combustion and post-combustion CCS technologies.
Cement-Oxy-n	A newly established cement production plant incorporating oxy-combustion and post-combustion CCS technologies.
Steel-DRI	A plant employing direct reduction of iron (DRI) technique using syngas derived from natural gas to produce steel.

Continued on next page

Table 2 – continued from previous page

Technology	Description
SteelDRI-CCS-n	A newly established plant utilising DRI technique with natural gas and equipped with CCS technology.
SteelDRI-CCS-r	An existing plant employing direct DRI technique with natural gas retrofitted with CCS technology.
SteelDRI-30H-n	A newly established plant employing DRI technique with a fuel blend of natural gas and 30% hydrogen.
SteelDRI-30H	An existing plant employing DRI technique that switched to a fuel blend of natural gas and 30% hydrogen.
SteelDRI-CCS-30H-n	A newly established plant utilising DRI technique that uses natural gas blended with 30% hydrogen and equipped with CCS technology.
SteelDRI-CCS-30H	An existing plant employing DRI technique and CCS that switched to a fuel blend of natural gas and 30% hydrogen.
SteelDRI-CCS-30H-r	An existing plant employing DRI technique fueled by natural gas blended with 30% hydrogen that is retrofitted with CCS technology.
Steel-BioGasif.-DR-n	A newly established steel production plant employing DRI technique and utilising a gasifier with biomass as feedstock for syngas production.
Steel-BioGasif.-DR-highCCS-n	A newly established steel production plant employing DRI technique and utilising a gasifier with biomass as feedstock for syngas production. The plant is equipped with CCS that contributes as a NET.
Steel-BioGasif.-DR-highCCS-r	An existing steel production plant employing DRI technique and utilising a gasifier with biomass as feedstock for syngas production. The plant is retrofitted with CCS technology that contributes as a NET.
Steel-BioGasif.-DR-highCCS-rg	An existing steel production plant utilising the DRI technique with CCS and retrofitted with a biomass gasifier to replace natural gas.
Steel-MOE	A facility utilising molten oxide electrolysis (MOE) approach for steel production, relying solely on electricity for the process.
Steel-EAF-scrap	A facility utilising electric arc furnace (EAF) technology for steel production, utilising steel scrap as feedstock and operating solely on electricity.
Steel-EW	A facility utilising electrowinning (EW) technology for steel production, relying solely on electricity for the process.
Refinery	A facility for crude oil refining.
Refinery-PCCS-r	An existing crude oil refinery retrofitted with CCS technology.
Refinery-PCCS-n	A newly constructed crude oil refinery equipped with CCS technology.
LNG Process	A facility specialising in natural gas liquefaction to produce LNG.
LNG Process - CCS - n	A newly established LNG production facility incorporating CCS technology.

Continued on next page

Table 2 – continued from previous page

Technology	Description
LNG Process - CCS -r	An existing LNG production facility retrofitted with CCS technology.
E-LNG	A facility specialising in natural gas liquefaction to produce LNG, with electrified refrigeration process.
Al-Smelter	A facility producing aluminum utilising an electrolyser connected to a CCGT plant.
Al-Smelter-CCS	A newly established aluminum production facility utilising an electrolyser connected to a CCGT plant equipped with CCS technology.
Al-Smelter-CCS-r	An existing aluminum production facility utilising an electrolyser connected to a CCGT plant that is retrofitted with CCS technology.
Al-Smelter-Grid-c	An existing aluminum production facility utilising an electrolyser disconnected from a CCGT plant and connected to the grid for power supply.
Al-Smelter-Grid	An existing aluminum production facility utilising an electrolyser connected directly to the grid for power supply.
Ammonia-HB	A facility producing ammonia using the Haber Bosch process.
AmmoniaBlue-r	An existing ammonia production facility utilising the Haber Bosch process retrofitted with CCS technology.
AmmoniaBlue-n	A newly established ammonia production facility utilising the Haber Bosch process equipped with CCS technology.
AmmoniaGreen	A facility producing ammonia using green hydrogen as feedstock.
HydrogenGrey	A facility producing hydrogen using conventional Steam Methane Reforming (SMR) process.
HydrogenBlue - n	A facility producing hydrogen using conventional SMR process equipped with CCS technology.
HydrogenBlue - r	An existing facility producing hydrogen using conventional SMR process retrofitted with CCS technology.
HydrogenGreen	A facility producing hydrogen using water electrolysis process.
SAF-HEFA-SPK	A facility utilising hydrogenation process with cooking oil/vegetable oil as feedstock to produce SAF.
SAF-PtL-Green	A process utilising green hydrogen and captured CO_2 in the Fischer Tropsch (FT) process for the production of SAF.

The Transport Sector Module

To incorporate the transport sector into the model, the formulation shown in equations 1 - 3 were integrated in the ESO-X model. The used nomenclature is detailed in Table 4. Equation 1 leverages various technologies to fulfill the demand for different modes of transport. The considered transport

modes encompass road vehicles (cars, buses and trucks) as well as maritime vessels for sea transport and airplanes for air transport. It is important to note the inclusion of a slack variable in equation 1 that is limited by equation 2. This addition is to relax equation 1 and is constrained by a very small fraction (0.0005%) of the transport demand for each mode. Equation 3 addresses transport emissions, which is subsequently incorporated into equation 6. This comprehensive representation of the transport sector in the model ensures a detailed consideration of various modes, technologies and associated emissions. The technologies used to meet the demand for each mode of transportation are outlined in Table 3 and Appendix .

$$\sum_{\text{itransp}} (\text{Ttype}_{\text{mo,itransp}} \times \text{trans}_{\text{itransp},a} \times \text{tDes}_{\text{itransp}}) = \text{TDem}_{\text{mo},a} - \text{slakT}_{\text{mo},a} \tag{1}$$

$$\text{slakT}_{\text{mo},a} \leq 0.000005 \times \text{TDem}_{\text{vehicles},a} \tag{2}$$

$$eTrans_{\text{itransp,a}} = tDes_{\text{itransp}} \times trans_{\text{itransp,a}} \times TTech_{\text{itransp,ems}} \tag{3}$$

$$pTrans_{\text{itransp,a}} = trans_{\text{itransp,a}} \times TTech_{\text{itransp,power}} \tag{4}$$

Table S3: List of technologies used in the transport sector segment of the ESO model [S12, 13, 14, 15, 16].

Technology	Description
Car-Gasoline	An internal combustion engine (ICE) vehicle designed for passenger transportation, powered by gasoline.
Car-Hybrid	An ICE vehicle designed for passenger transportation utilising gasoline and complimented with an electric motor.
Car-Electric	A passenger vehicle powered solely by an electric motor and rechargeable battery.
Car-Hydrogen	An ICE vehicle designed for passenger transportation, fueled by hydrogen gas.
Bus-Diesel	A mini bus equipped with an ICE running on diesel fuel.
Bus-Hybrid	A mini bus equipped with an ICE running on diesel fuel, complemented by an electric motor.
Bus-Electric	A mini bus powered by an electric motor and rechargeable battery.
Bus-Hydrogen	A mini bus equipped with an ICE running on hydrogen fuel.

Continued on next page

Table 3 – continued from previous page

Technology	Description
Truck-Diesel	A heavy-duty truck powered by an ICE running on diesel fuel for freight transportation.
Truck-Electric	A heavy-duty truck propelled solely by an electric motor and rechargeable battery.
Truck-Hydrogen	A heavy-duty truck equipped with an ICE running on hydrogen fuel.
Train-Diesel	A locomotive-powered train utilising an ICE running on diesel fuel for rail transportation.
Train-Electric	A train powered by electricity.
Train-Electric-r	A train formerly powered by a diesel locomotive, now operates using electricity.
Train-Hydrogen	A locomotive-powered train fueled by hydrogen gas.
Ship-HFO	A maritime vessel fueled by heavy fuel oil (HFO).
Ship-Ammonia-r	An existing maritime vessel retrofitted with an ammonia engine for propulsion, transitioning from conventional fuel to ammonia.
Ship-Ammonia	A maritime vessel powered by an ammonia engine.
Plane-JetFuel	A conventional aircraft powered by jet fuel for air transportation.
Plane-50SAF-fs	An existing aircraft transitioning from 100% jet fuel to a blend of 50% SAF and 50% jet fuel.
Plane-50SAF	A new aircraft fueled by a blend of 50% SAF and 50% jet fuel.

Carbon Dioxide Removal Technologies Module

In the ESO-X version utilised, only biomass-based power plant with CCS (BECCS) is employed for carbon emissions removal. A novel extension to the model includes the incorporation of direct air capture (DAC) and is introduced as an independent service linked to the grid. To meet the power requirements for operating DAC, equation 5 is employed to calculate the power demand based on the required levels of carbon removal. The extent of carbon removal is determined using equation 6. This enhancement broadens the model's capability to explore and optimise carbon removal strategies by integrating DAC alongside BECCS.

$$pDAC_{\text{DAC,a}} = eDAC_{\text{DAC,a}} \times \text{DTech}_{\text{PowerDAC}} \tag{5}$$

System Emissions Module

In this section of the model, modifications were made to incorporate the emissions of the transport sector and the emissions removed by DAC. The emissions removal by DAC is introduced as a free variable and is determined by the need for adjusting the system's emissions to meet the wanted target of carbon reduction. As presented in equation 6, the emissions removal ($eDAC_{\text{DAC,a}}$) compensates for any emissions from any sector. This adjustment enables a comprehensive consideration of both the transport sector emissions and the flexible emissions removal capability provided by DAC for the overall sectors in the model framework.

$$\sum_{ipc,c,t} e_{ipc,a,c,t}\text{WF}_{c,a} + \sum_{ii} eInd_{\text{ii,a}} + \sum_{i_{\text{transp}}} eTrans_{\text{itransp},a} + \sum_{i_{\text{DAC}}} eDAC_{\text{DAC,a}} \times \text{DTech}_{\text{emsDAC}} \leq SE_{\text{a}} \tag{6}$$

Power Requirement Module

To incorporate all the shown modifications above into the power demand, the constraint in the model was revised to include the demands from each sector individually (power, industry and transport) along with DAC as shown in equation 5. The assumption is that the power demand for transport is evenly distributed throughout the year and the same distribution applies to DAC. This refinement ensures a more accurate representation of power demand across various sectors and facilitates a more detailed understanding of sectoral power requirement.

$$\sum_{ipg} p2d_{ipg,a,c,t} + \sum_{ips} s2d_{ips,a,c,t} = SD_{c,t,a}(1+TL) - \text{slak}_{a,c,t} + \sum_{\text{DAC}} \frac{pDAC_{\text{DAC},a}}{8760} + \sum_{\text{itransp}} \frac{pTrans_{\text{itransp,a}}}{1000 \times 8760} + \sum_{ii,comm} \frac{pInd_{a,ii,comm}}{8760} \tag{7}$$

Table S4: Nomenclature used in the mathematical formulation of ESO-X model.

Type	Symbol	Description	Unit
set	a	Planning periods	years
	ii	Industrial Technologies	-
	ipc	Thermal technologies	-
	ipg	Generating technologies	-
	ips	Electricity storage technologies	-
	$itransp$	Transport technologies	-
	mo	Mode of transport (cars, bus, truck, train, ship and plane)	-
	$comm$	Commodity (Cement, Steel, Refinery, LNG, Aluminium, Ammonia, Hydrogen and SAF)	-
Parameter	$DTech_{DAC,}$	DAC technology features (emissions and power)	-
	$SD_{c,t,a}$	System demand for electricity	MWh/h
	SE_a	System emissions target	t-CO_2
	$tDes_{itransp}$	Unit size of $itransp$ technology	t-CO_2/year
	$TDem_{mo,a}$	Transportation demand	units of transport mode[1]
	TL	transmission losses	% of MW
	$TTech_{itransp,}$	Transport technology features (emissions and power)	-
	$Ttype_{mo,itransp}$	1 if $itransp$ can be used for mo, 0 otherwise	-
	$WF_{c,a}$	Demand weighing factor	-
variable	$eDAC_{DAC,a}$	Emissions removed by DAC	tCO_2/year
	$eInd_{ii,a}$	Industrial emissions	tCO_2/year
	$e_{ipc,a,c,t}$	Power emissions	t-CO_2
	$eTrans_{itransp,a}$	Carbon emissions by $itransp$	t-CO_2
	$p2d_{ipg,a,c,t}$	Power to demand	MW
	$pDAC_{DAC,a}$	Power requirement by DAC	MWh
	$pInd_{a,ii,comm}$	Power requirement by the industrial sector	MW
	$pTrans_{itransp,a}$	Power requirement by the transport sector	MW
	$s2d_{ips,a,c,t}$	Storage to demand	MW
	$slakT_{mo,a}$	Unmet transport demand	units of transport mode[1]
	$slak_{a,c,t}$	Unmet electricity demand	MW
	$trans_{itransp,a}$	Transportation by $itransp$ for mo variable	units of transport

[1] Except for ships, the value is in dead weight ton (DWT)

Modelling the UAE Energy System

To simulate the decarbonisation of the UAE economy, the industrial segment of the ESO model by Gazner [S5] was extended to incorporate additional technologies that were shown in Table 2. Moreover, the transport sector has been integrated into the model, as explained earlier. This section provides an overview of the costs and parameters applied in adapting the ESO model to the UAE context. The comprehensive structure of the model is shown in Figure 1 in the article.

System Parameters

Power Sector

To model the power sector in the UAE using ESO-X, an hourly demand profile was needed for the country. Since this level of granularity was not available, the profile shape was derived from a publication focused on the State of Qatar [S17]. The choice of Qatar as a reference was motivated by its geographical proximity to the UAE and its monthly demand profile that aligns closely with that of the UAE [S17, 18]. Additionally, both regions share comparable weather temperature variations [S19] and like the UAE, Qatar is an energy net-exporter [S20]. Given these similarities, the assumption that the UAE's hourly demand profile shape is similar to Qatar's was considered to be a reasonable approximation.

By utilising this assumption, the clustered demand profile for the UAE is illustrated in Figure 1, alongside wind and solar availability profiles extracted from Renewables Ninja [S21]. It is important to note that for solar availability, data were extracted from locations in Al Dhafra (western) region as vast land is available for solar farms and most of the existing solar capacity is in that region. Data for onshore wind were collected from regions near Sir Bani Yas, aligning with ongoing onshore wind projects in that area [S22]. This region was also observed to have the highest availability of onshore wind. With regards to offshore wind, data were extracted from seawater locations around the Emirate of Fujairah shore, where the highest wind availability was recorded there. In terms of imported electricity price, this price was assumed to be constant as neighbouring countries with existing interconnections (KSA and the Sultanate of Oman) operate based on fixed tariffs that depend on the amount of consumed electricity [S23, 24].

The costs, capacity and maximum deployment associated with power generation technologies in ESO-UAE were derived from ongoing investments if the data was available. Tables 5 and 6 present the data used in the ESO-UAE model for the power sector. For ancillary services to keep the grid stable at 50 Hz [S25], system inertia was kept at 39,000 MW.s, which is based on a blackout incident in the UAE [S26, 27]. The reserve capacity was found by trial and error and kept at 20% which was further confirmed by published data [S28]. Other relevant assumptions are listed in section .

Industrial Sector

To characterise the industrial sector in the UAE, this study expanded the ESO model to include the production of LNG, aluminum, ammonia and hydrogen. It is important to highlight that fuel switching was integrated into the model using the same formulation as technology retrofitting. Additionally, the DAC archetype technology considered in this model is from Climeworks. This technology utilises waste heat and can operate at low temperatures (100 °C) [S29]. Similar to the power sector, investment costs were utilised to represent the implemented technologies in the model when available. Tables 7 to 29 present the parameters associated with this segment of the model.

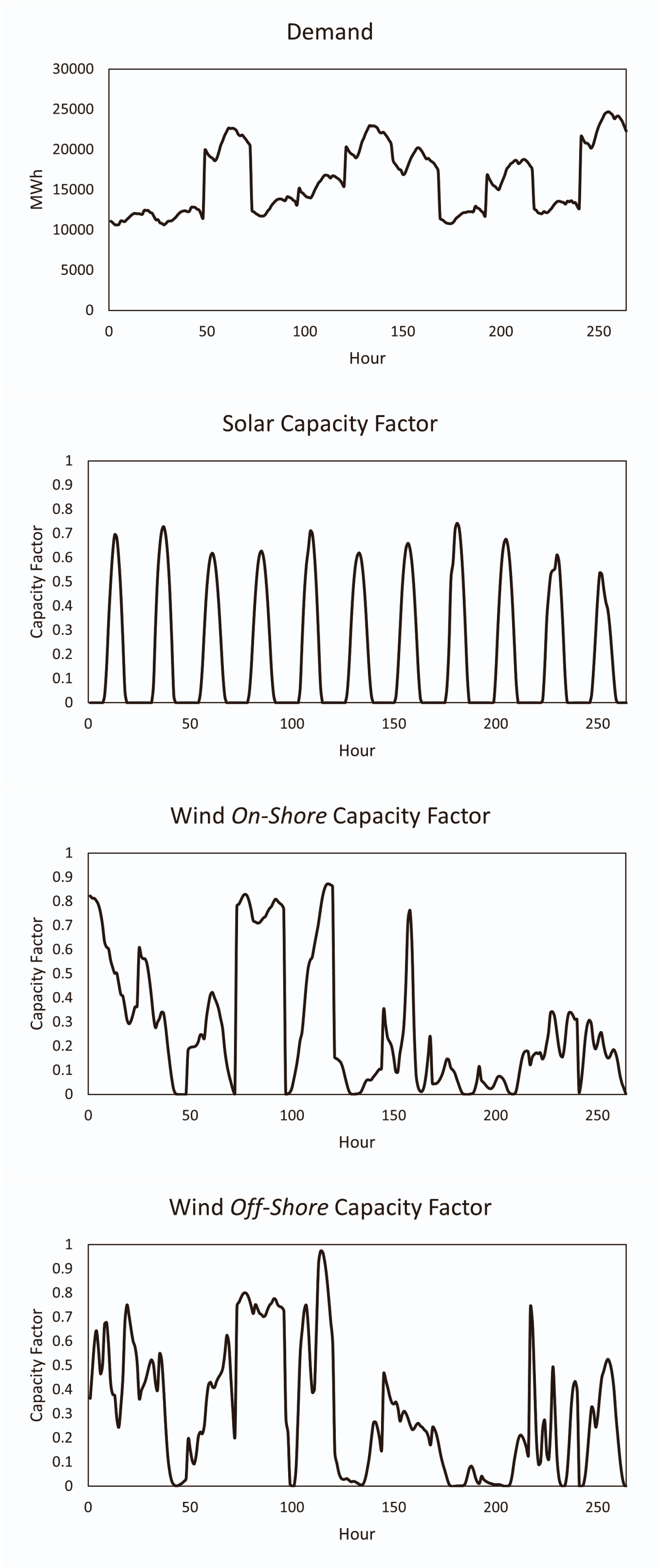

Figure S1: Clustered hourly 11 days of power demand along solar and wind capacity factors used in the ESO-UAE model.

Transport Sector

To characterise the transport sector in the UAE, this model considered road, sea and air transport. The existing capacity was derived from available data. Notably, OPEX and emissions for each transportation type were based on the average traveled distance. For shipping, the model employed deadweight tons (DWT) as units for vessels, rather than a single vessel unit. This decision was made considering the diverse sizes of vessels, aiming to identify which vessel types might need transformation.

Key Assumptions

1. **No New Diesel Generators**: It is assumed that no more diesel generators are built. This assumption is based on the existing generators in the Emirate of Sharjah, where new capacity has been utilising natural gas [S30].

2. **No Additional Interconnections with KSA and Oman**: It is assumed that no more electrical interconnections are built with KSA due to the frequency mismatch (KSA operates at 60 Hz [S31], while the UAE operates at 50 Hz [S25]). Similarly, it is assumed that no additional capacity is built with Oman and the existing capacity is sufficient.

3. **H_2-CCGT Build Rate and Operating Magnitude**: It is assumed that the maximum build rate for an H_2-CCGT is the same as that for a CCGT. Additionally, it is assumed that the cost magnitude of operating an H_2-CCGT (excluding fuel) increases at the same magnitude as the CAPEX relative to a conventional CCGT.

4. **Nuclear Power Plant Planning and Construction**: It is assumed that the planning and construction of nuclear power plants occur every 10 years, not during every planning period. This assumption is based on the Barakah nuclear power plant [S32].

5. **Biomass-Based Power Plant Size**: It is assumed that the size of biomass power plants (including BECSS-r) is 50 MW when domestically available biomass is used [S33, 34].

6. **Plant Lifespan**: It is assumed that all plants are new and will not retire during the modeled time periods. This assumption is based on the fact that most of the plants in the UAE are newly built.

7. **Demand Profile Shape**: It is assume that the shape of the demand profile is similar to that of Qatar. This assumption is made because the hourly demand profile for the UAE was not available.

8. **Unified Grid Assumption**: The UAE's electricity grid operates as an interconnected system, allowing for the transfer of power between emirates to ensure stability and reliability. While regional differences in energy demand, resource availability and infrastructure conditions exist across the UAE, the lack of detailed regional hourly demand profiles limits the granularity of spatial modeling in this study. For the purpose of this analysis, a unified grid approach is assumed, which enables the aggregation of demand and resource data into a single system-level model.

9. **Cooling Demand in the UAE**: It is assumed that the cooling demand in the UAE is predominantly electrified and is, therefore, inherently accounted for within the power sector modeling in this study. As a result, while the buildings sector is not explicitly modeled as a separate category, its energy demand is mostly driven by cooling requirements [S35] and is captured within the power sector's electricity consumption.

10. **Production Expansion of Steel, Cement and Aluminum**: It is assumed that the production of commodities does not expand. Specifically, in terms of steel, cement and aluminum, this assumption is based on the premise that the existing capacity was utilised for the construction of the Barakah nuclear power plant. Following the completion of this power plant, these commodities can be reallocated for the construction of various technologies during the transition period.

11. **Electrolyser-Produced Products Capacity Factor**: For products produced using electrolysers, 80% capacity factor is assumed.

12. **Emissions Reduction with 30% H_2 in Steel DRI**: By using 30% hydrogen in steel DRI, it is assumed that emissions are reduced by the amount of hydrogen that replaces natural gas [S7].

13. **Road Vehicles Retirement**: It is assumed that the retirement of road vehicles takes place within the first three planning periods. This assumption is based on the UAE regulation that prohibits the renewal of licenses for road vehicles after 15 years of use [S36].

14. **Hydrogen Vehicles Operating Expenditure**: It is assumed that the increase in fixed OPEX for hydrogen-fueled vehicles is proportional to the increase of hydrogen vehicles CAPEX relative to ICE vehicles CAPEX.

15. **Diesel Locomotive Capacity**: It is assumed a diesel locomotive has the capacity to carry 8,500 tons.

16. **Air and Sea Transport Emissions**: It is assumed that the UAE takes full responsibility for the emissions associated with these modes of transportation regardless of the travel direction.

17. **Vessel Utilisation**: It is assumed an annual utilisation rate of 65% for all the vessels in the UAE throughout the year [S37].

18. **Maritime Fleet Capacity Growth**: It is assumed that every 10 years, the capacity of the maritime fleet increases in alignment with the growth projected for the year 2030 [S38].

19. **Truck Fleet Growth**: It is assumed that future freight demand will be predominantly met by the railway fleet and the truck fleet will not experience significant growth.

20. **Public Transport Requirement**: It is assumed that the public transport requirement will be satisfied by the expansion of passenger railway services and the demand for buses will remain constant without substantial growth.

21. **Expansion of Non-Fuel Products:** It is assumed that the UAE will continue investing in the expansion of non-fuel products, such as advanced polymers, to diversify its economy beyond fossil fuel exports. This assumption is based on ongoing developments in companies like Borouge [S39], which focus on converting fossil fuels into high-value products that are not directly linked to emissions.

22. **Economic Growth Assumption:** The study assumes economic growth through planned production expansions in oil, gas, ammonia and hydrogen, reflecting the UAE's strategy to sustain industrial activity amid the global energy transition. These planned expansions indicate an effort to balance energy sector growth with decarbonization goals. Given the variation in carbon reduction targets among different countries, continued fossil fuel demand is expected in the near-to-medium term. For instance, India has set a net-zero target for 2070 [S40], suggesting a prolonged reliance on fossil fuel imports. This reinforces the assumption that global energy markets will still require fossil-based energy exports in the coming decades, supporting the UAE's strategy to maintain production capacity while integrating lower-carbon alternatives.

The UAE and Climate Change

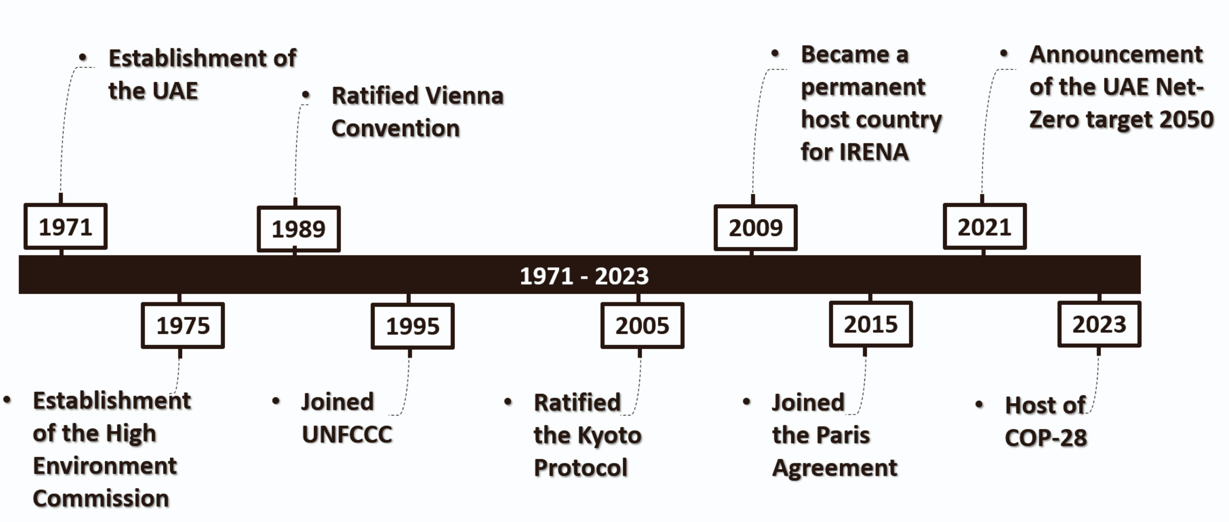

Figure S2: A summary of the UAE's environmental evolution during the 1971-2023 period. [S41, 42].

Environmental conservation has been a fundamental value in the UAE since its establishment. Founded by H.H. Sheikh Zayed bin Sultan Al Nahyan in 1975, the High Environment Commission led the charge in environmental preservation efforts, evolving into what is known today as the Federal Environment Agency [S42, 43]. More recently, the Emirate of Abu Dhabi has emerged as a hub for renewable energy initiatives, hosting the International Renewable Energy Agency (IRENA) in Masdar City, a designated economic zone focused on renewable energy investments. Additionally, the UAE has pioneered a new energy strategy incorporating nuclear and solar power in its energy mix, marking a significant departure from traditional energy sources [S41].

Building on this foundation, the UAE became the first country in the Middle East and North Africa (MENA) to commit to a net-zero target by 2050. Although not mandated by the UNFCCC due to its status as a non-Annex 1 country, the UAE voluntarily chose to monitor and reduce its GHG emissions.

From an economic standpoint, the UAE is an oil net-exporter, yet it has long recognised the importance of diversifying its revenue streams [S44]. Since 2008, the country has pursued a vision of transitioning to a non-oil-based economy, resulting in a notable 17% increase in non-oil revenue in 2022 alone [S45, 46]. With a firm commitment to carbon neutrality, the UAE has set ambitious targets for clean ammonia, hydrogen and SAF production. These targets include producing 3 mt/yr of blue or green ammonia by 2030, establishing a blue or green hydrogen production capacity of 15 mt/yr by 2050 and reaching 5 mt/yr of SAF production by 2050 [S47, 48, 49].

While transitioning towards alternative fuels, the UAE also plans to increase its oil production to 250 mt/yr by 2030 [S50]. Initiatives such as the development of the Hail and Ghasha fields for natural gas processing and LNG export aim to elevate production levels to 27 mt/yr by 2030 from the current 6 mt/yr [S51]. Given these ambitious targets, it becomes imperative to consider how the UAE can navigate this transition while ensuring economic stability, particularly considering its role as a fuel supplier to other nations such as Japan and India, both of which have their own net-zero targets [S52, 53, 40, 54].

Building upon the insights that explored the impact of fuel prices and the effect of a CDR target on technology deployment rates, similar findings were observed in studies by Ganzer et al. when the industrial sector was modeled alongside the power sector [S5, 55]. As current literature lacks studies on a net-zero UAE, it is imperative to comprehensively model the interplay between the power, industrial and transport sectors to gain a wholistic understanding of the UAE's transition pathway to net-zero.

Method

The ESO framework is utilised to derive the results based on the UAE's published emissions reduction plan [S56]. The plan outlines a trajectory aiming to decrease current emissions level from 200 mt-CO_2 to 185 mt-CO_2 by the year 2030 and ultimately reaching net-zero emissions by 2050. This trajectory of emissions served as the fundamental input within the ESO framework and is as visualised in Figure 3.

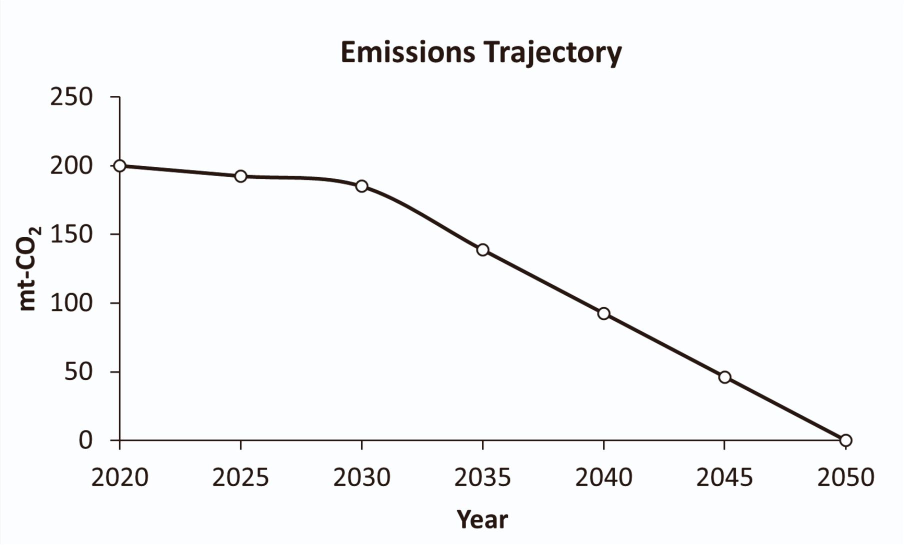

Figure S3: Emissions target used in ESO-UAE model.

In terms of maximum allowable capacity for each technology, detailed descriptions can be found in Table 6. It is important to note that this study exclusively considers commercially available technologies.

As for industrial targets, the analysis assumes an increase in oil production capacity to 250 mt/yr by 2030 from the current level of 160 mt/yr and this capacity is assumed to be maintained until 2050. Similarly, for LNG, the production capacity is projected to rise from 6 mt/yr to 27 mt/yr by 2030 and is assumed to remain constant until 2050. These projections are based on ongoing projects mentioned earlier.

Table S5: Power generation technology costs and parameters in the ESO-UAE model.

Technology	CAPEX (£/kW)	Unit Size (MW/unit)	Build Rate (Unit(s)/year)	Capacity (MW)	Ref.
Nuclear	3604	1345	0.4	2690	[S57, 58]
Coal	1540	500	0	0	[S59]
Bio	2250	500[1]	0.6	0	[S60, 61, 62, 63]
CCGT	721	600	5	31200	[S64, 59]
CCGT-PostCCS	1531	750	1.2	0	
CCGT-PostCCS-r	811	600	1.2	0	[S65]
BECCS	3826	500	1.2	0	[S60, 63]
BECCS-r	1827	500[1]	1.2	0	[S65]
Wind-Onshore	981	20	1	0	[S60, 61, 62, 63]
Wind-Offshore	2047	50	1	0	[S60, 61, 62, 63]
Solar	616	600	1	2400	[S64, 66]
InterImp	1001	500	0	1500	[S67]
PHSto	1211	125	2	0	[S68]
Oil	803	75	0	825	[S64]
H_2-CCGT	1550	600	1	0	[S69]

[1] 50 MW if the scenario is considering domestic biomass only.

Table S6: Power generation maximum allowable capacity in the ESO-UAE model.

Technology	Maximum Capacity
Nuclear	Unlimited or zero depending on the scenario
Coal	None - The area is rich with natural gas. The country used to have a coal capacity for a short period of time. This plant was converted to operate on natural gas [S59].
Bio	Unlimited or constrained based on nationally available biomass
CCGT	Unlimited
CCGT-PostCCS	Unlimited
CCGT-PostCCS-r	Unlimited
BECCS	Unlimited or zero depending on the scenario
BECCS-r	Unlimited or constrained based on nationally available biomass
Wind-Onshore	Unlimited
Wind-Offshore	Unlimited
Solar	Unlimited
InterImp	None assuming the existing capacity is enough. This is based on the fact that Abu Dhabi operated as reserve capacity for the Gulf region
PHSto	Limited based on dam availability in the UAE [S70]
Oil	None as recent ones have been converted into natural gas power plants
H_2-CCGT	Unlimited or 0 depending on the scenario

Table S7: Cost input for cement production used technologies in the ESO-UAE model [S5, 71, 72].

Technology	Product (X)	CAPEX (m£/mt-X)	OPEX (m£/mt-X)
Cement Plant	Cement	250	41
CementPCCS-r	Cement	12	66
CementPCCS-n	Cement	263	66
CementOxy-r	Cement	114	37
CementOxy-n	Cement	296	37

Table S8: Existing capacity for cement production used in the ESO-UAE model [S73].

Technology	Product (X)	Capacity (mt-X/year)
Cement	Cement	30
CementPCCS-r	Cement	
CementPCCS-n	Cement	
CementOxy-r	Cement	
CementOxy-n	Cement	

Table S9: Emissions and their sources for cement production technologies used in the ESO-UAE model [S5, 74, 75].

Technology	Emissions (t-CO_2/mt-X)	Source
Cement	591500	
CementPCCS-r	59150	Capture is from clinker flue gas
CementPCCS-n	59150	Capture is from clinker flue gas
CementOxy-r	88000	
CementOxy-n	88000	

Table S10: Cost input for steel production technologies used in the ESO-UAE model [S5, 7, 76, 77, 78, 79, 80, 81, 82, 83].

Technology	Product (X)	CAPEX (m£/mt-X)	OPEX (m£/mt-X)
Steel-DRI	Steel	204	148
SteelDRI-CCS-n	Steel	253	150
SteelDRI-CCS-r	Steel	49	150
SteelDRI-30H-n	Steel	204	148
SteelDRI-30H	Steel		148
SteelDRI-CCS-30H-n	Steel	253	148
SteelDRI-CCS-30H	Steel		148
SteelDRI-CCS-30H-r	Steel	49	150
Steel-BioGasif.-DR-n	Steel	3071828	215
SteelDRI-BioGasif.-CCS-n	Steel	3071877	217
SteelDRI-BioGasif.-CCS-r	Steel	3071662	217
SteelDRI-BioGasif.-CCS-rg	Steel	3071613	217
Steel-MOE	Steel	1000	601
Steel-EAF-scrap	Steel	115	390
Steel-EW	Steel	548	601

Table S11: Existing capacity and power requirement for steel production used in the ESO-UAE model [S5, 84, 85, 86, 87].

Technology	Product (X)	Capacity (mt-X/year)	Power (TWh/mt-X)
Steel-DRI	Steel		
SteelDRI-CCS-n	Steel	2	
SteelDRI-CCS-r	Steel		
SteelDRI-30H-n	Steel		
SteelDRI-30H	Steel		
SteelDRI-CCS-30H-n	Steel		
SteelDRI-CCS-30H	Steel		
SteelDRI-CCS-30H-r	Steel		
SteelDRI-BioGasif.-n	Steel		
SteelDRI-BioGasif.-CCS-n	Steel		
SteelDRI-BioGasif.-CCS-r	Steel		
SteelDRI-BioGasif.-CCS-rg	Steel		
Steel-MOE	Steel		4000
Steel-EAF-scrap	Steel	2	563
Steel-EW	Steel		2583

Table S12: Emission sources for steel production using various technologies in the ESO-UAE model [S6, 7].

Technology	Emissions ($t\text{-}CO_2$/mt-X)	Source
Steel-DRI	804000	Emissions from DRI and pre-treatment reactors
SteelDRI-CCS-n	356700	Emissions are from pre-treatment reactors and 90% is captured from the DRI.
SteelDRI-CCS-r	356700	Emissions are from pre-treatment reactors and 90% is captured from the DRI.
SteelDRI-30H-n	739000	Emissions from DRI and pre-treatment reactors
SteelDRI-30H	739000	Emissions from DRI and pre-treatment reactors
SteelDRI-CCS-30H-n	350200	Emissions are from pre-treatment reactors and 90% is captured from the DRI.
SteelDRI-CCS-30H	350200	Emissions are from pre-treatment reactors and 90% is captured from the DRI.
SteelDRI-CCS-30H-r	350200	Emissions are from pre-treatment reactors and 90% is captured from the DRI.
SteelDRI-BioGasif.-n	-430699	The negative emissions result from utilizing biomass, primarily from 90% of DRI capture, while accounting for supply chain emissions, gasifier emissions and pretreatment reactors.
SteelDRI-BioGasif.-CCS-n	-430699	The negative emissions result from utilizing biomass, primarily from 90% of DRI capture, while accounting for supply chain emissions, gasifier emissions and pretreatment reactors.
SteelDRI-BioGasif.-CCS-r	-430699	The negative emissions result from utilizing biomass, primarily from 90% of DRI capture, while accounting for supply chain emissions, gasifier emissions and pretreatment reactors.
SteelDRI-BioGasif.-CCS-rg	-430699	The negative emissions result from utilizing biomass, primarily from 90% of DRI capture, while accounting for supply chain emissions, gasifier emissions and pretreatment reactors.
Steel-MOE	-	-
Steel-EAF-scrap	-	-
Steel-EW	-	-

Table S13: Cost input for oil-refining technologies used in the ESO-UAE model [S5, 88, 89].

Technology	Product (X)	CAPEX (m£/mt-X)	OPEX (m£/mt-X)
Refinery	Refined-Oil	94	108
Refinery-PCCS-r	Refined-Oil	31	137
Refinery-PCCS-n	Refined-Oil	124	137

Table S14: Existing capacity for oil-refining used in the ESO-UAE model [S90].

Technology	Product (X)	Capacity (mt-X/year)
Refinery	Refined-Oil	169
Refinery-PCCS-r	Refined-Oil	
Refinery-PCCS-n	Refined-Oil	

Table S15: Emission sources for oil-refining using various technologies in the ESO-UAE model [S91, 92].

Technology	Emissions ($t\text{-}CO_2$/mt-X)	Source
Refinery	300587	Emissions are from upstream and downstream operations
Refinery-PCCS-r	81378	90% capture of downstream emissions
Refinery-PCCS-n	81378	90% capture of downstream emissions

Table S16: Cost input for LNG technologies used in the ESO-UAE model [S8, 93, 94, 95, 96].

Technology	Product (X)	CAPEX (m£/mt-X)	OPEX (m£/mt-X)
LNGprocess	LNG	141	18
LNGprocess-PCCS-r	LNG	141	18
LNGprocess-PCCS-n	LNG	788	18
LNGprocess-Electric	LNG	647	4

Table S17: Existing capacity and power requirement for LNG production used in the ESO-UAE model [S97, 98].

Technology	Product (X)	Capacity (mt-X/year)	Power (MWh/mt-X)
LNGprocess	LNG	8	
LNGprocess-highPCC-r	LNG		
LNGprocess-highPCC-n	LNG		
LNGprocess-Electric	LNG		330000

Table S18: Emission sources for LNG production using various technologies in the ESO-UAE model [S8, 99].

Technology	Emissions ($t\text{-}CO_2$/mt-X)	Source
LNGprocess	600000	
LNGprocess - CCS - n	372000	Post-combustion capture from gas turbine for LNG refrigeration. The remaining emissions are from natural gas pre-treatment and exploration
LNGprocess - CCS - r	372000	Post-combustion capture from gas turbine for LNG refrigeration. The remaining emissions are from natural gas pre-treatment and exploration.
E-LNG	360000	Emissions are from upstream and exploration.

Table S19: Cost input for Aluminium technologies used in the ESO-UAE model [S59, 100].

Technology	Product (X)	CAPEX (m£/mt-X)	OPEX (m£/mt-X)
Al-Smelter	Aluminium	1563	229
Al-Smelter-CCS-n	Aluminium	3183	259
Al-Smelter-CCS-r	Aluminium	1743	259
Al-Smelter-Grid-c	Aluminium		27
Al-Smelter-Grid	Aluminium	123	27

Table S20: Existing capacity and power requirement for Aluminium production used in the ESO-UAE model [S10].

Technology	Product (X)	Capacity (mt-X/year)	Power (TWh/mt-X)
Al-Smelter	Aluminium	4.5	
Al-Smelter-CCS-n	Aluminium		
Al-Smelter-CCS-r	Aluminium		
Al-Smelter-Grid-c	Aluminium		14000
Al-Smelter-Grid	Aluminium		14000

Table S21: Emission sources for Aluminium production using various technologies in the ESO-UAE model [S101].

Technology	Emissions ($t\text{-}CO_2$/mt-X)	Source
Al-Smelter	5437385	Processing and CCGT plant
Al-Smelter-CCS	543739	90% Capture from CCGT power plant
Al-Smelter-CCS-r	543739	90% Capture from CCGT power plant
Al-Smelter-Grid-c		
Al-Smelter-Grid		

Table S22: Cost input for Ammonia technologies used in the ESO-UAE model [S102, 103].

Technology	Product (X)	CAPEX (m£/mt-X)	OPEX (m£/mt-X)
Ammonia-HB	Ammonia	374	9
AmmoniaBlue-r	Ammonia	169	19
AmmoniaBlue-n	Ammonia	543	19
AmmoniaGreen	Ammonia	2576	

Table S23: Existing capacity and power requirement for Ammonia production used in the ESO-UAE model [S103, 104, 105].

Technology	Product (X)	Capacity (mt-X/year)	Power (MWh/mt-X)
Ammonia-HB	Ammonia	2	83333
AmmoniaBlue-r	Ammonia		361111
AmmoniaBlue-n	Ammonia		361111
AmmoniaGreen	Ammonia		10500000

Table S24: Emission sources for Ammonia production using various technologies in the ESO-UAE model [S103].

Technology	Emissions (t-CO_2/mt-X)	Source
Ammonia-HB	2350000	
AmmoniaBlue-r	120000	95% capture
AmmoniaBlue-n	120000	95% capture
AmmoniaGreen		

Table S25: Cost input for Hydrogen technologies used in the ESO-UAE model [S106, 107, 108, 109].

Technology	Product (X)	CAPEX (m£/mt-X)	OPEX (m£/mt-X)
HydrogenGrey	Hydrogen	4003	524
HydrogenBlue-r	Hydrogen	2685	586
HydrogenBlue-n	Hydrogen	6688	586
HydrogenGreen	Hydrogen	5239	191

Table S26: Existing capacity and power requirement for Hydrogen production used in the ESO-UAE model [S109, 110].

Technology	Product (X)	Capacity (mt-X/year)	Power (TWh/mt-X)
HydrogenGrey	Hydrogen	0.3	
HydrogenBlue-r	Hydrogen		
HydrogenBlue-n	Hydrogen		
HydrogenGreen	Hydrogen		56300

Table S27: Emission sources for Hydrogen production using various technologies in the ESO-UAE model [S103, 107].

Technology	Emissions ($t\text{-}CO_2$/mt-X)	Source
HydrogenGrey	9305786	
HydrogenBlue - n	987989	90% capture from SMR furnace flue gas. Capture is based on MEA chemical absorption
HydrogenBlue - r	987989	90% capture from SMR furnace flue gas. Capture is based on MEA chemical absorption
HydrogenGreen	-	

Table S28: Cost input for SAF technologies used in the ESO-UAE model [S11].

Technology	Power (MWh/mt-X)	CAPEX (m£/mt-X)	OPEX (m£/mt-X)
SAF-HEFA-SPK		90	220
SAF-PtL-Green	17461574	1600	121

Table S29: Cost input for DAC technologies used in the ESO-UAE model [S111].

Technology	Power (MWh/$t\text{-}CO_2$)	CAPEX (£/$t\text{-}CO_2$)	OPEX (£/$t\text{-}CO_2$)
DAC	2.03	389	213

Table S30: Cost input for road transport vehicles used in the ESO-UAE model [S12, 13, 14, 16, 112, 113, 114, 115, 116, 117, 118, 119, 120, 121, 122, 123, 124, 125, 126, 127, 128].

Vehicle	CAPEX (£/vehicle)	OPEX (£/vehicle)
Car-Gasoline	21213	2420
Car-Hybrid	40658	2040
Car-Electric	44240	1490
Car-Hydrogen	49214	2049
Bus-Diesel	39708	8150
Bus-Hybrid	24855	8230
Bus-Electric	71100	2130
Bus-Hydrogen	531250	20778
Truck-Diesel	48765	6532
Truck-Electric	142200	
Truck-Hydrogen	400000	7149

Table S31: Capacity and power requirement for road transport vehicles used in the ESO-UAE model [S129, 130, 131, 132, 133, 134].

Vehicle	Capacity (vehicles)	Power (kWh/vehicle)
Car-Gasoline	3600000	
Car-Hybrid		252
Car-Electric	39000	4258
Car-Hydrogen		
Bus-Diesel	99490	
Bus-Hybrid	3189	2536
Bus-Electric	100	29484
Bus-Hydrogen		
Truck-Diesel	237427	
Truck-Hybrid		
Truck-Electric		29193
Truck-Hydrogen		

Table S32: Emission from road transport vehicles in the ESO-UAE model [S12, 13, 14, 117, 128, 135].

Vehicles	Emissions (t-CO_2/vehicle per year)
Car-Gasoline	4
Car-Hybrid	2.4
Car-Electric	
Car-Hydrogen	
Bus-Diesel	31
Bus-Hybrid	28
Bus-Electric	
Bus-Hydrogen	
Truck-Diesel	19
Truck-Electric	
Truck-Hydrogen	

Table S33: Cost input for train transport used in the ESO-UAE model [S136, 137, 138, 139, 140].

Train Type	CAPEX (£/train)	OPEX (£/train)
Train-Diesel	1537114	213111
Metro	1537114	100328
Train-Electric-r		100328
Train-Electric	1537114	100328
Train-Hydrogen	81840000	5360588

Table S34: Capacity and power requirement for road transport vehicles used in the ESO-UAE model [S15, 135, 137, 138, 141, 142, 143].

Train Type	Capacity (Trains)	Emissions (t-CO_2/train)	Power (GWh/train per year)
Train-Diesel	45	705	
Metro	39		190
Train-Electric-r			190
Train-Electric			190
Train-Hydrogen			

Table S35: Cost input for maritime transportation used in the ESO-UAE model [S144, 145, 146, 37, 147, 148, 149].

Ship Type	CAPEX (£/DWT[1])	OPEX (£/DWT)
Ship-HFO	755	153
Ship-Ammonia-r	387	304
Ship-Ammonia	1142	304

Table S36: Maritime transportation capacity and emissions data used in the ESO-UAE model [S37, 146, 147, 148, 149, 150, 151].

Ship Type	Capacity (DWT)	Emissions (t-CO_2/DWT)
Ship-HFO	1711700	0.66
Ship-Ammonia-r		
Ship-Ammonia		

Table S37: Cost input for aviation used in the ESO-UAE model [S152, 153, 154, 155].

Plane Type	CAPEX (£/plane)	OPEX (£/plane)
Plane-JetFuel	110600000	20206321
Plane-50SAF-fs		30974777
Plane-50SAF	110600000	30974777

Table S38: Airplane capacity and emissions data used in the ESO-UAE model [S151, 154, 156, 157, 158].

Plane Type	Capacity (planes)	Emissions (t-CO_2/plane per year)
Plane-JetFuel	385	93736
Plane-50SAF-fs		46868
Plane-50SAF		46868

Table S39: Fuel prices used in the ESO-UAE model.

Fuel	Price	Units	Note	Ref.
Natural Gas	8	£/MWh		[S159, 160]
Gasoline	0.7	£/L		[S161]
Diesel	0.7	£/L		[S162]
JetFuel	0.4	£/L		[S163]
Heavy Fuel Oil	11	£/L		[S164, 165]
Uranium	5	£/MWh		[S166]
Biomass	5	£/MWh	Domestic	[S167, 33]
Biomass	20	£/MWh	Imported	[S168]
Hydrogen	2	£/kg	Averaged levelised cost of green and blue hydrogen	
Cooking Oil	43	£/ton		

Bibliography

[S1] C. F. Heuberger, I. Staffell, N. Shah, and N. Mac Dowell, "Quantifying the value of ccs for the future electricity system," *Energy & Environmental Science*, vol. 9, no. 8, pp. 2497–2510, 2016.

[S2] C. F. Heuberger, E. S. Rubin, I. Staffell, N. Shah, and N. Mac Dowell, "Power capacity expansion planning considering endogenous technology cost learning," *Applied Energy*, vol. 204, pp. 831–845, Oct. 2017.

[S3] C. F. Heuberger, P. K. Bains, and N. Mac Dowell, "The ev-olution of the power system: A spatio-temporal optimisation model to investigate the impact of electric vehicle deployment," *Applied Energy*, vol. 257, p. 113715, Jan. 2020.

[S4] ADNOC, "ADNOC to Build World-Scale Blue Ammonia Project," May 24 2021. Available online at `https://www.adnoc.ae/news-and-media/press-releases/2021/adnoc-to-build-world-scale-blue-ammonia-project`.

[S5] C. Ganzer, *Pathways to net zero for power and industry in the United Kingdom*. PhD thesis, Imperial College London, Centre for Environmental Policy, Oct 2022. Available online at `http://hdl.handle.net/10044/1/100342`.

[S6] Z. Fan and S. J. Friedmann, "Low-carbon production of iron and steel: Technology options, economic assessment, and policy," *Joule*, vol. 5, no. 4, pp. 829–862, 2021. Available online at `https://doi.org/10.1016/j.joule.2021.02.018`.

[S7] T. Astoria, G. Hughes, and N. Mizutani, "MIDREX NG™ with H_2 Addition: Moving from natural gas to hydrogen in decarbonizing ironmaking," tech. rep., MIDREX Technologies, Inc., 3735 Glen Lake Drive, Suite 400, Charlotte, North Carolina 28208 USA, March 2022. Available online at `https://www.midrex.com/wp-content/uploads/2022/03/MIDREX-NG-with-H2-Addition-1.pdf`.

[S8] IEAGHG, "Techno-economic evaluation of co_2 capture in lng plants," Tech. Rep. 2019-07, October 2019. Available online at `http://tinyurl.com/4mf4b65w`.

[S9] R. Skiebe, "All-electric lng maximizes process control in the arctic," *Gas Processing & LNG*, March/April 2020. Available online at https://assets.siemens-energy.com/siemens/assets/api/uuid:88cb7d7c-548a-4d8d-b46e-93540d20ddd2/gp-mar-apr2020-electric-lng-article.pdf.

[S10] A. Sultan, "Power Generation." Available online at https://primary.world-aluminium.org/processes/power-generation/.

[S11] McKinsey & Company and World Economic Forum, "Scaling sustainable aviation fuel today for clean skies tomorrow," 2020. Available online at https://www.mckinsey.com/~/media/mckinsey/industries/travel%20transport%20and%20logistics/our%20insights/scaling%20sustainable%20aviation%20fuel%20today%20for%20clean%20skies%20tomorrow/clean-skies-for-tomorrow.pdf.

[S12] NimbleFins, "Average MPG for cars UK (2024)," 2024. Available online at https://www.nimblefins.co.uk/cheap-car-insurance/average-mpg#:~:text=Petrol%20cars%20average%20around%2036,the%20equivalent%20of%20132%20MPGe.

[S13] J. O'Dea, "Electric vs. diesel vs. natural gas: Which bus is best for the climate?," July 19 2018. Available online at https://blog.ucsusa.org/jimmy-odea/electric-vs-diesel-vs-natural-gas-which-bus-is-best-for-the-climate/#:~:text=Fuel%20efficiency&text=Fuel%20efficiencies%20used%20in%20this,a%2090%20percent%20charging%20efficiency.

[S14] M. T. Parts, "What fuel economy (mpg) does a lorry get?." Available online at https://mwtruckparts.co.uk/what-fuel-economy-mpg-does-a-lorry-get#:~:text=Whilst%20modern%20diesel%20cars%20can,over%2033t%20was%207.6mpg.

[S15] J. Wang and H. A. Rakha, "Electric train energy consumption modeling," *Applied Energy*, vol. 193, pp. 346–355, 2017. Available online at https://doi.org/10.1016/j.apenergy.2017.02.058.

[S16] S. Dermühl and U. Riedel, "A comparison of the most promising low-carbon hydrogen production technologies," *Fuel*, vol. 340, p. 127478, 2023. Available online at https://doi.org/10.1016/j.fuel.2023.127478.

[S17] "Generation, analysis, and applications of high resolution electricity load profiles in qatar," *Journal of Cleaner Production*, vol. 183, pp. 527–543, May 2018. Available online at https://doi.org/10.1016/j.jclepro.2018.02.084.

[S18] S. Taqvi, A. Almansoori, A. Maroufmashat, and A. Elkamel, “Utilizing rooftop renewable energy potential for electric vehicle charging infrastructure using multi-energy hub approach,” *Energies*, vol. 15, p. 9572, 12 2022. DOI: 10.3390/en15249572.

[S19] W. Spark, “Compare the climate and weather in doha and Abu Dhabi,” 2021. Available online at https://weatherspark.com/compare/y/105083~105360/Comparison-of-the-Average-Weather-in-Doha-and-Abu-Dhabi.

[S20] International Energy Agency, “Qatar.”

[S21] S. Pfenninger and I. Staffell, “Renewables ninja,” 2016. Available online at https://www.renewables.ninja/.

[S22] Masdar, “Masdar launches UAE’s first utility scale wind project with breakthrough low wind speed innovation,” October 5 2023. Available online at http://tinyurl.com/3jw67ts2.

[S23] Global Petrol Prices, “Electricity prices in saudi arabia.” Available online at https://www.globalpetrolprices.com/Saudi-Arabia/electricity_prices/.

[S24] Mazoon Electricity Company, “Tariff detail.” Available online at https://mzec.nama.om/en-us/Pages/tarifftype.aspx.

[S25] ADE, “Ac voltages & frequencies per country.” Available online at https://ade-power.com/info/worldwide-voltage-frequency-list.

[S26] Emirates Green Building Council, “Benchmarking program.” Available online at https://emiratesgbc.org/technical-programs/benchmarking-program/.

[S27] ArabianPost, “Power cut plunges Dubai mall into darkness – retail.” Available online at https://thearabianpost.com/power-cut-plunges-dubai-mall-into-darkness-retail/#:~:text=Dubai%20Mall%2C%20one%20of%20the,the%20mall%20in%20the%20dark.

[S28] MEED, “UAE to diversify energy supply,” 2011. Available online at https://www.meed.com/uae-to-diversify-energy-supply/ Published on 16 March 2011.

[S29] Climeworks, “Direct air capture: our technology to capture co_2.” Available online at https://climeworks.com/direct-air-capture#:~:text=Once%20sucked%20in%2C%20it%20passes,for%20a%20cup%20of%20tea!

[S30] “Hamriyah combined-cycle independent power project, Sharjah, UAE.” Power Technology, October 2023. Available online at https://www.power-technology.com/projects/hamriyah-power-project-sharjah-uae/?cf-view.

[S31] S. Alqahtani, A. Shaher, A. Garada, and L. Cipcigan, “Impact of the high penetration of renewable energy sources on the frequency stability of the saudi grid,” *Electronics*, vol. 12, no. 6, 2023. Available online at https://www.mdpi.com/2079-9292/12/6/1470.

[S32] S.-B. Kim and J.-H. Keppler, “The barakah nuclear power plants, the united arab emirates,” 2013. Available online at https://www.oecd-nea.org/ndd/workshops/wpne/presentations/docs/4_2_KIM_%20Barakah%20presentation.pdf.

[S33] M. T. Ashraf, C. Fang, T. Bochenski, I. Cybulska, A. Alassali, A. Sowunmi, R. Husain Farzanah, G. Brudecki, T. Chaturvedi, S. Haris, J. Schmidt, and M. Thomsen, “Estimation of bioenergy potential for local biomass in the united arab emirates,” *Emirates Journal of Food and Agriculture*, vol. 28, p. 1, 01 2016. DOI: 10.9755/ejfa.2015-04-060.

[S34] I. Mubeen and A. Buekens, “Chapter 14 - energy from waste: Future prospects toward sustainable development,” in *Current Developments in Biotechnology and Bioengineering* (S. Kumar, R. Kumar, and A. Pandey, eds.), pp. 283–305, Elsevier, 2019. Available online at https://www.sciencedirect.com/science/article/pii/B9780444640833000142.

[S35] M. H. Elnabawi, E. Saber, and L. Bande, “Passive building energy saving: Building envelope retrofitting measures to reduce cooling requirements for a residential building in an arid climate,” *Sustainability*, vol. 16, no. 2, p. 626, 2024.

[S36] A. Ahmed, “UAE no place for old cars,” *Gulf News*, August 2008. Available online at https://gulfnews.com/uae/transport/uae-no-place-for-old-cars-1.127376.

[S37] E. Lindstad, D. Polić, A. Rialland, I. Sandaas, and T. Stokke, “Decarbonizing bulk shipping combining ship design and alternative power,” *Ocean Engineering*, vol. 266, p. 112798, 2022. Available online at https://doi.org/10.1016/j.oceaneng.2022.112798.

[S38] S. Khan, “AD ports to buy eight bulk carriers and crude tankers in deals worth $260m,” *The National News*, May 2023. Available online at http://tinyurl.com/3hnm27x6.

[S39] Borouge, “Committed to sustainability: Borouge’s approach to environmental and social responsibility,” 2023. Borouge is a leading petrochemical company providing innovative polyolefin solutions, committed to sustainability, circular economy initiatives, and addressing global challenges such as CO2 emissions, food protection, and clean water distribution.

[S40] Ministry of Environment, Forest and Climate Change, “Net zero emissions target.” Posted On: 03 AUG 2023 5:04PM by PIB Delhi, 2023. Available online at https://pib.gov.in/PressReleaseIframePage.aspx?PRID=1945472#:~:text=India%2C%20at%20the%2026th%20session,achieve%20net%20zero%20by%202070.

[S41] The United Arab Emirates' Government Portal, "The UAE's response to climate change." Available online at https://u.ae/en/information-and-services/environment-and-energy/climate-change/theuaesresponsetoclimatechange.

[S42] Emirates News Agency - WAM, "Zayed considered environment as," April 2005. Available online at https://wam.ae/ru/details/1395227411559.

[S43] United Arab Emirates, "Federal law no.7 of 1993 establishing federal environment authority." UNEP - LEAP, 1993. Available online at https://leap.unep.org/en/countries/ae/national-legislation/federal-law-no7-1993-establishing-federal-environment-authority.

[S44] LSE Middle East Centre, "Export composition and economic growth in the UAE." in collaboration with the American University of Sharjah. Available online at https://www.lse.ac.uk/middle-east-centre/research/collaboration-programme/2019-20/athanasia-kalaitzi LSE PI: Dr. Athanasia Kalaitzi, Co-PI: Dr. Samer Kherfi, Duration: August 2019 – October 2021.

[S45] Government of Abu Dhabi, *The Abu Dhabi Economic Vision 2030*. 2008. Available online at https://www.actvet.gov.ae/en/Media/Lists/ELibraryLD/economic-vision-2030-full-versionEn.pdf.

[S46] R. Uppal, "UAE's non-oil foreign trade jumps 17% in 2022," February 2023. Available online at https://www.reuters.com/markets/uaes-non-oil-foreign-trade-jumps-17-2022-2023-02-06/.

[S47] United Arab Emirates, "UAE Net Zero 2050," 2023. Available online at https://u.ae/en/information-and-services/environment-and-energy/climate-change/theuaesresponsetoclimatechange/uae-net-zero-2050.

[S48] United Arab Emirates Ministry of Energy & Infrastructure, "UAE Hydrogen Leadership Roadmap," tech. rep., November 2021. Available online at https://u.ae/en/about-the-uae/strategies-initiatives-and-awards/strategies-plans-and-visions/environment-and-energy/national-hydrogen-strategy.

[S49] United Arab Emirates Government, "General Policy for Sustainable Aviation Fuel," June 2024. Available online at http://tinyurl.com/prr48a3k.

[S50] A. D. Paola, "UAE accelerates plan to increase its oil production capacity," 2022. Available online at https://www.bloomberg.com/news/articles/2022-09-19/uae-accelerates-plan-to-increase-its-oil-production-capacity?leadSource=uverify%20wall.

[S51] GlobalData, "Oil & gas field profile: Hail & ghasha conventional gas field, UAE." Updated July 30, 2023. Available online at https://www.offshore-technology.com/data-insights/oil-gas-field-profile-hail-ghasha-conventional-gas-field-uae/.

[S52] T. J. Times, "Uniting paths for carbon neutrality," November 1 2021. Available online at https://www.japantimes.co.jp/2021/11/01/special-supplements/uniting-paths-carbon-neutrality/ Accessed on: January 4, 2024.

[S53] C. Klein, "Crude oil imports from the u.a.e. to japan fy 2022, by oil type," June 2023. Available online at https://www.statista.com/statistics/761790/japan-crude-oil-imports-from-uae-by-oil-type/#:~:text=In%20the%20fiscal%20year%202022,at%20around%2017.7%20million%20kiloliters.

[S54] India Briefing from Dezan Shira & Associates, "India, UAE commence crude oil trade using local currency settlement (lcs) framework," Available online at http://tinyurl.com/y9t5sust.

[S55] C. Ganzer and N. Mac Dowell, "Pathways to net zero for power and industry in the UK," *International Journal of Greenhouse Gas Control*, vol. 125, p. 103887, 2023. Available online at https://doi.org/10.1016/j.ijggc.2023.103887.

[S56] Ministry of Climate Change and Environment, UAE, "Accelerating action towards a green, inclusive, determined contribution for the UAE: Third update of second nationally determined contribution for a sustainable and resilient economy," 2023. Available online at http://www.moccae.gov.ae.

[S57] A. S. Matthew Martin, "Abu dhabi said to revive debt plan for first nuclear power plant," September 2015. Available online at https://shorturl.at/KMW02.

[S58] "Nuclear power in the united arab emirates." Updated January 2024, 2024. Available online at https://world-nuclear.org/information-library/country-profiles/countries-t-z/united-arab-emirates.aspx.

[S59] PJSC, "Hassyan power complex, dubai, UAE." Available online at https://www.power-technology.com/projects/hassyan-clean-coal-project-dubai/?cf-view&cf-closed.

[S60] European Commission and Directorate-General for Climate Action and Directorate-General for Energy and Directorate-General for Mobility and Transport, A. De Vita, P. Capros, L. Paroussos, K. Fragkiadakis, P. Karkatsoulis, L. Höglund-Isaksson, W. Winiwarter, P. Purohit, A. Gómez-Sanabria, P. Rafaj, L. Warnecke, A. Deppermann, M. Gusti, S. Frank, P. Lauri, F. Fulvio,

A. Florou, M. Kannavou, N. Forsell, T. Fotiou, P. Siskos, P. Havlík, I. Tsiropoulos, S. Evangelopoulou, P. Witzke, M. Kesting, N. Katoufa, I. Mitsios, G. Asimakopoulou, and T. Kalokyris, *EU reference scenario 2020 – Energy, transport and GHG emissions – Trends to 2050*. Publications Office, 2021. doi/10.2833/35750.

[S61] M. . Company, *Transformation of Europe's power system until 2050*. 2010. Available online at https://www.mckinsey.com/~/media/mckinsey/dotcom/client_service/epng/pdfs/transformation_of_europes_power_system.ashx.

[S62] International Renewable Energy Agency, "Renewable power generation costs in 2022," tech. rep., International Renewable Energy Agency, Abu Dhabi, 2023. Available online at https://www.irena.org/Publications/2023/Aug/Renewable-Power-Generation-Costs-in-2022.

[S63] Department of Business, Energy & Industrial Strategy, "Electricity generation costs 2020," tech. rep., Department of Business, Energy & Industrial Strategy, 2020. Available online at https://www.gov.uk/government/publications/beis-electricity-generation-costs-2020.

[S64] United Arab Emirates Ministry of Cabinet Affairs, "Agriculture environment and energy." Federal Competitiveness and Statistics Centre, 2020. Available online at https://fcsc.gov.ae/en-us/Pages/Statistics/Statistics-by-Subject.aspx.

[S65] IEAGHG, "Effects of plant location on the costs of co_2 capture," Technical Report 2018-04, IEAGHG, April 2018.

[S66] NS Energy, "Noor Abu Dhabi Solar Power Project." Available online at https://www.nsenergybusiness.com/projects/noor-abu-dhabi-solar-power-project/.

[S67] Gulf Cooperation Council Interconnection Authority, "The interconnection project." Available online at https://www.gccia.com.sa/P/the_interconnection_project/55.

[S68] Power Technology, "Hatta Pumped Storage Hydropower Plant, UAE." Available online at https://www.power-technology.com/projects/hatta-pumped-storage-hydropower-plant-uae/?cf-view Accessed on: Insert Access Date.

[S69] S. Öberg, M. Odenberger, and F. Johnsson, "Exploring the competitiveness of hydrogen-fueled gas turbines in future energy systems," *International Journal of Hydrogen Energy*, vol. 47, no. 1, pp. 624–644, 2022. Available online at https://doi.org/10.1016/j.ijhydene.2021.10.035.

[S70] S. A. Alnaqbi, S. Alasad, H. Aljaghoub, A. H. Alami, M. A. Abdelkareem, and A. G. Olabi, "Applicability of hydropower generation and pumped hydro energy storage in the middle east and north africa," *Energies*, vol. 15, no. 7, p. 2412, 2022. Available online at https://doi.org/10.3390/en15072412.

[S71] B. Standard, "Ultratech invests $101.1 mn in UAE-based rakwct, acquires 29.39% share." Business Standard, January 27 2024. Available online at https://t.ly/-2jk9.

[S72] "Ccs from cement production." Available online at https://www.ctc-n.org/technologies/ccs-cement-production Accessed: 27th January 2024.

[S73] Global Cement Magazine, "Total installed capacity of cement plants in the united arab emirates (UAE) 2019, by producer." Statistic as Excel data file, 2019.

[S74] P. S. Fennell, S. J. Davis, and A. Mohammed, "Decarbonizing cement production," *Joule*, vol. 5, no. 6, pp. 1305–1311, 2021. Available online at https://doi.org/10.1016/j.joule.2021.04.011.

[S75] T. Hills, D. Leeson, N. Florin, and P. Fennell, "Carbon capture in the cement industry: Technologies, progress, and retrofitting," *Environmental Science & Technology*, vol. 50, no. 1, pp. 368–377, 2016. Available online at https://doi.org/10.1021/acs.est.5b03508 PMID: 26630247.

[S76] M. Watts, "United iron & steel company to invest $138m in UAE rolling mill," *MEED*, April 15 2014. Available online at https://www.meed.com/united-iron-steel-company-to-invest-138m-in-uae-rolling-mill/.

[S77] International Energy Agency, "Iron and steel technology roadmap: Towards more sustainable steelmaking." This publication is subject to specific restrictions that limit its use and distribution. The terms and conditions are available online at www.iea.org/t&c/. Source: IEA. All rights reserved.

[S78] G. E. Monitor, "Ghc emirates steel industries Abu Dhabi plant," 2023. Available online at https://www.gem.wiki/GHC_Emirates_Steel_Industries_Abu_Dhabi_plant.

[S79] Element Energy Limited, "Industrial carbon capture business models," tech. rep., The Department for Business, Energy and Industrial Strategy, Suite 1, Bishop Bateman Court, Thompson's Lane, Cambridge CB5 8AQ, October 2018. Supported by: Element Energy Limited, Tel: 01223 852499.

[S80] S. S. Tam, "Gasification plant cost and performance optimization," 5 2002. Available online at https://www.osti.gov/biblio/837331.

[S81] P. E. Duarte, "HYL direct reduction technology: Adaptations for the indian market," *HYL*. Available online at https://www.energiron.com/wp-content/uploads/2019/05/2004-HYL-Direct-Reduction-Technology-Adaptations-for-the-Indian-Market.pdf.

[S82] Y. Cao, Z. Gao, J. Jin, H. Zhou, M. Cohron, H. Zhao, H. Liu, and W. Pan, "Synthesis gas production with an adjustable H_2/CO ratio through the coal gasification process: Effects of coal ranks and methane addition," *Energy & Fuels*, vol. 22, no. 3, pp. 1720–1730, 2008. 10.1021/ef7005707.

[S83] M. De Santis, A. Di Donato, T. Kempken, T. Hauck, M. Draxler, A. Sormann, P. Queipo, W. Szulc, D. Croon, J.-T. Ghenda, C. Wang, J.-C. Pierret, and J. Borlee, "Green steel for europe investment needs," tech. rep., March 2021. This project has received funding from the European Union under grant agreement NUMBER — 882151 — GREENSTEEL.

[S84] WAM, "Emirates steel's exports expand to 56 international markets," Mar. 2022. Available online at https://www.wam.ae/en/details/1395303030757#:~:text=Emirates%20Steel%20has%20a%20capacity,diversification%20by%20boosting%20its%20exports. Accessed on January 28, 2024.

[S85] COSASTEEL, "Steel company in UAE." Available online at https://www.cosasteel.com/steel-company-uae/ Accessed on March 18, 2022.

[S86] Emirates Steel Arkan, "Our journey: A history of looking to the future." Available online at https://www.emiratessteelarkan.com/our-journey/#:~:text=Its%20capacity%20of%20500%2C000%20metric,and%20the%20UAE's%20steel%20industry.

[S87] A. Rauwerdink, "Steel production through electrolysis: Impacts for electricity consumption." VP, Business Development, October 2019.

[S88] "ESFC: International investment, global engineering and financial consulting." Website, 2024. Available online at https://esfcinvestment.com/ Accessed on January 28, 2024.

[S89] D. Johansson, P. Åke Franck, and T. Berntsson, "CO_2 capture in oil refineries: Assessment of the capture avoidance costs associated with different heat supply options in a future energy market," *Energy Conversion and Management*, vol. 66, pp. 127–142, 2013. Available online at https://doi.org/10.1016/j.enconman.2012.09.026.

[S90] Organization of the Petroleum Exporting Countries, "UAE facts and figures." Website, 2024. Available online at https://www.opec.org/uae-facts-and-figures Accessed on January 28, 2024.

[S91] Y. Saba, "UAE oil giant raises climate goal ahead of key UN summit," *Reuters*, July 2023. Available online at https://www.reuters.com/article/us-emirates-oil-climatechange-idUSKBN2F20QK Accessed on January 28, 2024.

[S92] J. van Straelen, F. Geuzebroek, N. Goodchild, G. Protopapas, and L. Mahony, “CO_2 capture for refineries, a practical approach,” in *Proceedings of the 9th International Conference on Greenhouse Gas Control Technologies (GHGT-9)*, (Amsterdam, The Netherlands), Shell Global Solutions, Shell Global Solutions, 2008.

[S93] Q. Zou, C. Yi, K. Wang, X. Yin, and Y. Zhang, “Global LNG market: supply-demand and economic analysis,” *IOP Conference Series: Earth and Environmental Science*, vol. 983, p. 012051, mar 2022. Available online at https://doi.org/10.1088%2F1755-1315%2F983%2F1%2F012051.

[S94] Staff Writer, “ADNOC awards $80 million contract for Hail and Ghasha gas development,” *Oil & Gas Middle East*, Jan 2023. Available online at https://www.oilandgasmiddleeast.com/news/adnoc-awards-80-million-contract-for-hail-and-ghasha-gas-development.

[S95] B. Songhurst, “LNG plant cost reduction 2014–18,” tech. rep., The Oxford Institute for Energy Studies, 2019. https://www.oxfordenergy.org/publications/lng-plant-cost-reduction-2014-18/.

[S96] European Technology Platform for Zero Emission Fossil Fuel Power Plants, “The costs of CO_2 capture, transport and storage,” tech. rep., Advisory Council of the European Technology Platform for Zero Emission Fossil Fuel Power Plants, Year.

[S97] S&P Global Commodity Insights, “FEATURE: ADNOC’s new Fujairah LNG project seeks to capitalize on global thirst for energy,” *SP Global Commodity Insights*, Jul 2022. Available online at https://t.ly/DNrtK.

[S98] “Chapter 5 - natural gas liquefaction cycle enhancements and optimization,” in *Handbook of Liquefied Natural Gas* (S. Mokhatab, J. Y. Mak, J. V. Valappil, and D. A. Wood, eds.), pp. 229–257, Boston: Gulf Professional Publishing, 2014. Available online at https://doi.org/10.1016/B978-0-12-404585-9.00005-2.

[S99] Timera Energy, “The value impact of LNG carbon emissions,” *Timera Energy*, Mar 2021. Available online at https://timera-energy.com/the-value-impact-of-lng-carbon-emissions/ Accessed on March 29, 2021.

[S100] K. Singh, “Vedanta aluminium sets capex target of $300 m for current fiscal,” *Businessline*, May 2021. Available online at https://www.thehindubusinessline.com/companies/vedanta-aluminium-sets-capex-target-of-300-m-for-current-fiscal/article34557268.ece Updated - May 18, 2021.

[S101] “Climate and carbon footprint.” Available online at https://aluminium.fr/en/climate-and-carbon-footprint/.

[S102] GlobalData, “Asia to lead global ammonia capacity additions.” News Analysis, September 2022. Available online at https://www.offshore-technology.com/news/asia-to-lead-global-ammonia-capacity-additions/.

[S103] International Energy Agency (IEA), “The future of hydrogen assumptions annex,” YEAR. Available online at https://iea.blob.core.windows.net/assets/a02a0c80-77b2-462e-a9d5-1099e0e572ce/IEA-The-Future-of-Hydrogen-Assumptions-Annex.pdf.

[S104] A. Puri-Mirza, “Production nitrogen content of ammonia in UAE by type 2010-2018.” Statista, 2021. Available online at https://www.statista.com/statistics/761200/uae-nitrogen-content-of-ammonia-production-by-type/.

[S105] I. Sen, “Shareholders sign taziz blue ammonia agreement,” *MEED*, January 2023. Available online at https://www.meed.com/shareholders-sign-agreement-for-taziz-blue-ammonia-facility.

[S106] “Methodology and specifications guide: Global hydrogen & ammonia.” Available online at www.spglobal.com/commodityinsights Latest update: October 2023.

[S107] IEAGHG, “Techno-Economic Evaluation of SMR Based Standalone (Merchant) Hydrogen Plant with CCS,” technical report, IEAGHG, 2017. Available online at https://www.ieaghg.org/docs/General_Docs/2017-02.pdf.

[S108] Department for Energy Security and Net Zero and Department for Business, Energy & Industrial Strategy, “Hydrogen supply chain: Evidence base.” GOV.UK, November 30 2018. Available online at https://www.gov.uk/government/publications/hydrogen-supply-chain-evidence-base.

[S109] P. Fragiacomo and M. Genovese, “Numerical simulations of the energy performance of a pem water electrolysis based high-pressure hydrogen refueling station,” *International Journal of Hydrogen Energy*, vol. 45, no. 51, pp. 27457–27470, 2020. Available online at https://doi.org/10.1016/j.ijhydene.2020.07.007.

[S110] J. Benny, “Site preparation under way for first phase of multi-billion dollar projects at ta’ziz,” November 4 2022. Available online at https://t.ly/0n2mq.

[S111] M. Sendi, M. Bui, N. Mac Dowell, and P. Fennell, “Geospatial analysis of regional climate impacts to accelerate cost-efficient direct air capture deployment,” *One Earth*, vol. 5, no. 10, pp. 1153–1164, 2022. Available online at https://doi.org/10.1016/j.oneear.2022.09.003.

[S112] Seez, "Average car listing price in the united arab emirates in 2018 and 2019 (in emirati dirhams)," 2020, March 1. Available online at https://www.statista.com/statistics/1156189/uae-average-car-listing-price/.

[S113] P. Bazaar, "What is the cost of car insurance in Dubai?." Available online at https://www.policybazaar.ae/what-is-the-cost-of-car-insurance-in-dubai-ciart/#:~:text=How%20Much%20Does%20Car%20Insurance,driving%20history%2C%20driving%20experience%20etc.

[S114] H. Zhang, W. Chen, and W. Huang, "Times modelling of transport sector in China and USA: Comparisons from a decarbonization perspective," *Applied Energy*, vol. 162, pp. 1505–1514, 2016. Available online at https://doi.org/10.1016/j.apenergy.2015.08.124.

[S115] Statista, "Electric vehicles - united arab emirates." Available online at https://www.statista.com/outlook/mmo/electric-vehicles/united-arab-emirates.

[S116] D. Cars, "Used hybrid cars for sale in UAE." Available online at https://www.dubicars.com/uae/used/hybrid-cars.

[S117] Phys.org, "Hybrid cars – pros and cons," January 19 2006. Available online at https://phys.org/news/2006-01-hybrid-cars-pros-cons.html#:~:text=Hybrids%20are%20the%20most%20gasoline,to%2060%20mpg%20(claimed).

[S118] J. Fossdyke, "Toyota mirai hydrogen UK pricing." motor1.com, May 5 2021. Available online at https://uk.motor1.com/news/505291/toyota-mirai-hydrogen-uk-pricing/.

[S119] "Toyota coaster toyota coaster 2023 diesel 4.2l." Available online at https://www.dubicars.com/2024-toyota-coaster-toyota-coaster-2023-diesel-42l-528354.html Updated: 28 Jan, 2024.

[S120] Sustainable Bus Editorial, "Major fuel cell bus order for solaris from rvk cologne: 15 solaris hydrogen on delivery," March 13 2020. Available online at https://t.ly/1qrzp.

[S121] L. Collins, "Europe's largest ever order for hydrogen buses awarded to poland's solaris after €272m tender," September 12 2023. Available online at https://rb.gy/rwg2ub.

[S122] R. Lydall, "Won't get fuelled again! london electric buses powered by 'world-first' rapid charging technology," October 28 2022. Available online at https://www.standard.co.uk/news/transport/london-buses-electric-rapid-charging-bexleyheath-b1035299.html#:~:text=The%20buses%20cost%20about%20%C2%A3,diesel%20or%20hybrid%20double%20decker.

[S123] T. Nguyen, “Australia’s first electric bus hits the market! 2022 joylong e6 priced above ford transit, mercedes sprinter, toyota hiace commuter and volkswagen crafter minibus,” June 3 2022. Available online at https://rb.gy/cfkkji.

[S124] S. Visawanathan, “Best commercial vehicles in UAE 2021-22: Price, specifications, mileage, colors, images,” August 16 2021. Available online at https://www.cars24.com/ae/blog/best-commercial-vehicles-in-uae/.

[S125] Toyota, “Proace verso.” Available online at https://www.toyota.co.uk/new-cars/proace-verso.

[S126] S. Ranganathan, “Hybrid buses costs and benefits,” Available online at https://www.eesi.org/files/eesi_hybrid_bus_032007.pdf.

[S127] C. Barnet, “Tesla opens order book for its semi to UK buyers,” May 16 2022. Available online at https://www.commercialmotor.com/product-news/article/tesla-opens-order-book-its-semi-uk-buyers.

[S128] dubizzle, “Buy & sell cars online in all cities (UAE), UAE.” Available online at https://uae.dubizzle.com/motors/used-cars/.

[S129] D. Hawley, “How much does it cost to charge an ev?,” 2022, September 20. Available online at https://www.jdpower.com/cars/shopping-guides/how-much-does-it-cost-to-charge-an-ev#:~:text=Most%20EVs%20can%20generally%20travel,and%20cost%20%2410%2D%2414.

[S130] T. Moloughney, “2023 toyota prius prime: Insideevs 70 mph range test.” InsideEVs, April 5 2023. Available online at https://insideevs.com/reviews/660293/2023-toyota-prius-prime-range-test/#:~:text=In%20addition%20to%20the%20great,of%2025%20miles%20per%20charge.

[S131] S. B. Editorial, “Electric bus range, focus on electricity consumption. a sum-up,” November 28 2023. Available online at https://www.sustainable-bus.com/news/electric-bus-range-electricity-consumption/.

[S132] Tesla, “Semi: The future of trucking is electric.” Available online at https://www.tesla.com/en_gb/semi#:~:text=With%20less%20than%202%20kWh,miles%20on%20a%20single%20charge.

[S133] Ministry of Energy Infrastructure, “Updated UAE energy strategy 2050,” tech. rep., July 2030.

[S134] S. T., “Automotive fleet market size - UAE,” September 6 2021. Available online at https://askwonder.com/research/automotive-fleet-market-size-uae-5h1c3e9m8.

[S135] United States Environmental Protection Agency (EPA), “Greenhouse gas emissions from a typical passenger vehicle,” 2023. Available online at https://www.epa.gov/greenvehicles/greenhouse-gas-emissions-typical-passenger-vehicle Last updated on August 28, 2023.

[S136] R. Technology, “Etihad rail, united arab emirates (UAE),” September 8 2022. Available online at https://www.railway-technology.com/projects/etihad-rail/?cf-view.

[S137] A. Talaiekhozani, O. Ghaffarpasand, M. R. Talaie khozani, N. Neshat, and B. Eydivandi, “Evaluation of emission inventory of air pollutants from railroad and air transportation in isfahan metropolitan in 2016,” *Journal of Air Pollution and Health*, vol. 2, pp. 1–18, 03 2017.

[S138] MEED Editorial, “ADNOC axes plan for shah sulphur pipeline,” May 5 2010. Available online at https://www.meed.com/adnoc-axes-plan-for-shah-sulphur-pipeline/.

[S139] mydubaistay, “Dubai metro time table.” Available online at http://www.mydubaistay.com/en/dubai-metro-routes.aspx.

[S140] R. Gopalan, M. Ravibabu, and S. Sahu, “Alternative approach to costing on indian railways: Linking outputs and expenses to activity centres,” *Asian Transport Studies*, vol. 6, p. 100001, 2020. Available online at https://doi.org/10.1016/j.eastsj.2020.100001.

[S141] Global Railway Review, “Etihad rail boosts its fleet size with order for 38 emd locomotives,” February 27 2020. Available online at https://www.globalrailwayreview.com/news/97440/etihad-rail-38-locomotives-progress-rail/.

[S142] A. Business, “Fifty new Dubai metro trains set to be deployed,” November 8 2020. Available online at https://www.arabianbusiness.com/industries/transport/454266-fifty-new-dubai-metro-trains-set-to-be-deployed#:~:text=Seats%20in%20the%20new%20trains,643%20riders%20to%20696%20riders.

[S143] Mitsubishi Heavy Industries Technical Review, “The Dubai metro, the world’s longest fully automated metro network,” vol. 49, June 2012. Available online at https://www.mhi.co.jp/technology/review/pdf/e492/e492001.pdf.

[S144] Made-in-China, “Vessel price,” 2023.

[S145] J. Atchison, “Retrofitting vessels for ammonia fuel: New technical study from grieg star,” March 21 2023. Available online at https://rb.gy/wufpwp.

[S146] D. Shafran, “How much fuel does a cargo ship use? ship fuel consumption explained with examples,” 2023. Available online at https://maritimepage.com/fuel-consumption-how-much-fuel-cargo-ship-use/#:~:text=For%20example%2C%20a%

20cargo%20ship%20with%20a%20capacity%20of%2050%2C000,also%20increase%20fuel%20consumption%20rates. Updated on November 16, 2023.

[S147] J. Dawson, L. Pizzolato, S. Howell, L. Copland, and M. Johnston, "Temporal and spatial patterns of ship traffic in the canadian arctic from 1990 to 2015 + supplementary appendix 1: Figs. s1–s7 (see article tools)," *ARCTIC*, vol. 71, p. 15, 02 2018. DOI: 10.14430/arctic4698.

[S148] SeaPlace, "Ship maintenance cost: How can owners reduce it?." Available online at https://www.seaplace.es/maintenance-cost-how-can-owners-reduce-it/ Accessed in 2023.

[S149] "Sea route & distance," 2023.

[S150] UNCTAD, "Maritime profile: United arab emirates," 2022. Available online at https://unctadstat.unctad.org/CountryProfile/MaritimeProfile/en-GB/784/index.html.

[S151] U.S. Energy Information Administration (EIA), "Carbon dioxide emissions coefficients."

[S152] Pilot Institute, "From gliders to jumbos: How much do airplanes cost?." Available online at https://pilotinstitute.com/how-much-do-airplanes-cost/ Posted on February 16, 2023.

[S153] M. Fioriti, V. Vercella, and N. Viola, "Cost-estimating model for aircraft maintenance," *Journal of Aircraft*, vol. 55, no. 4, pp. 1564–1575, 2018. Available online at https://doi.org/10.2514/1.C034664.

[S154] Epic Flight Academy, "How fast do commercial planes fly?." Available online at https://epicflightacademy.com/flight-school-faq/how-fast-do-commercial-planes-fly/#:~:text=FAQ%20%C2%BB%20Careers%2C%20General%20FAQs%20%C2%BB,%3B%20547%E2%80%93575%20mph.

[S155] Fly Emirates, "Emirates aircraft cover 432 million kilometres across the globe in six months," July 28 2016. Available online at https://rb.gy/utnn0l.

[S156] PlaneSpotters.NET, "Emirates fleet details and history." Available online at https://www.planespotters.net/airline/Emirates.

[S157] PlaneSpotters.NET, "Etihad airways fleet details and history." Available online at https://www.planespotters.net/airline/Etihad-Airways.

[S158] PlaneSpotters.NET, "Air arabia fleet details and history." Available online at https://www.planespotters.net/airline/Air-Arabia.

[S159] T. Robinson, "ADNOC natural gas, lng ipo seen as 'highly feasible' amid broader expansion plans," February 9 2023. Available online at https://rb.gy/xqc1yw.

[S160] MEED Editorial, “UAE purchases gas from Dolphin pipeline at reduced rate,” May 16 2008. Available online at https://www.meed.com/uae-purchases-gas-from-dolphin-pipeline-at-reduced-rate/.

[S161] “United arab emirates gasoline prices,” 2023. Available online at https://www.globalpetrolprices.com/United-Arab-Emirates/gasoline_prices/.

[S162] “United arab emirates diesel prices,” 2023. Available online at https://www.globalpetrolprices.com/United-Arab-Emirates/diesel_prices/.

[S163] Jet-A1-Fuel.com, “Jet a1 price united arab emirates.” Available online at https://jet-a1-fuel.com/price/united-arab-emirates.

[S164] H. N. Psaraftis and C. A. Kontovas, “Speed models for energy-efficient maritime transportation: A taxonomy and survey,” *Transportation Research Part C: Emerging Technologies*, vol. 26, pp. 331–351, 2013. Available online at https://doi.org/10.1016/j.trc.2012.09.012.

[S165] LiveBunkers, “Heavy fuel oil (HFO).” Available online at https://livebunkers.com/heavy-fuel-oil-hfo#:~:text=Density%3A,m3%20to%20facilitate%20efficient%20centrifuging.

[S166] World Nuclear Association, “Economics of nuclear power.” Updated August 2022, 2022. Available online at http://www.world-nuclear.org/information-library/economics-of-nuclear-power.aspx.

[S167] W. E. Forum, “Dubai has just increased the cost of landfill. can it hit zero waste by 2030?,” February 9 2022.

[S168] Argus, “Argus biomass markets,” tech. rep., 2023. Available online at https://www.argusmedia.com/-/media/Files/sample-reports/argus-biomass-markets.ashx.
